# Cross-sectional study of prevalence and correlates of fear of falling in the older people in residential care in India: the Hyderabad Ocular Morbidity in Elderly Study (HOMES)

**DOI:** 10.1136/bmjopen-2023-080973

**Published:** 2024-05-28

**Authors:** Srinivas Marmamula, Thirupathi Reddy Kumbham, Satya Brahmanandam Modepalli, Navya Rekha Barrenkala, Jill Elizabeth Keeffe, David S Friedman

**Affiliations:** 1 Allen Foster Community Eye Health Research Centre, Gullapalli Pratibha Rao International Centre for Advancement of Rural Eye care, L V Prasad Eye Institute, Hyderabad, Telangana, India; 2 School of Optometry and Vision Science, University of New South Wales, Sydney, New South Wales, Australia; 3 Wellcome Trust / Department of Biotechnology India Alliance, L V Prasad Eye Institute, Hyderabad, Telangana, India; 4 Harvard Medical School Department of Ophthalmology, Massachusetts Eye and Ear, Boston, MA, USA

**Keywords:** aging, epidemiology, geriatric medicine, public health

## Abstract

**Objective:**

To report the prevalence and risk factors for the fear of falling (FOF) among older individuals living in residential care facilities in India.

**Design:**

Cross-sectional study.

**Setting:**

Homes for the aged centres in Hyderabad, India.

**Participants:**

The study included individuals aged ≥60 years from homes for the aged centres. The participants underwent a comprehensive eye examination in make-shift clinics setup in homes. Trained investigators collected the personal and demographic information of the participants and administered the Patient Health Questionnaire-9 and Hearing Handicap Inventory for Elderly questionnaire in the vernacular language. FOF was assessed using the Short Falls Efficacy Scale. The presence of hearing and visual impairment in the same individual was considered dual sensory impairment (DSI). A multiple logistic regression analysis was done to assess the factors associated with FOF.

**Primary outcome measure:**

FOF.

**Results:**

In total, 867 participants were included from 41 homes for the aged centres in the analyses. The mean (±SD) age of the participants was 74.2 (±8.3) years (range 60–96 years). The prevalence of FOF was 56.1% (95% CI 52.7% to 59.4%; n=486). The multivariate analysis showed that those with DSI had eleven times higher odds of reporting FOF than those with no impairment (OR 11.14; 95% CI 3.15 to 41.4.) Similarly, those with moderate depression had seven times higher odds (OR 6.85; 95% CI 3.70 to 12.70), and those with severe depression had eight times higher odds (OR 8.13; 95% CI 3.50 to 18.90) of reporting FOF. A history of falls in the last year was also associated with increased odds for FOF (OR 1.52; 95% CI 1.03 to 2.26).

**Conclusion:**

FOF is common among older individuals in residential care in India. Depression, falling in the previous year and DSI were strongly associated with FOF. A cross-disciplinary approach may be required to address FOF among the older people in residential care in India.

**STRENGTHS AND LIMITATIONS OF THIS STUDY**

- Hyderabad Ocular Morbidity in Elderly Study (HOMES) is a cross-sectional study that included a large number of older people in residential care facilities in an urban region in India.
- All participants underwent comprehensive assessments including visual acuity measurement, hearing assessment, depression, mobility and other assessments.
- A multiple logistic regression analysis including visual impairment, hearing impairment and dual sensory impairment as covariates was done to assess the independent predictors of fear of falling in the older population.
- The hearing assessment was based on a questionnaire, and this might have underestimated the prevalence of lower grades of hearing impairments.
- The results of this study can only be generalised to the older population in residential care settings in urban regions in India.

## Introduction

The proportion of older people (people aged 60 years and older) in India’s population is on the rise, and it is expected to reach 20% of the total population by 2050.^1^ This demographic shift will dramatically change the nature of healthcare in India, and research on healthy ageing among the older population is needed. Such research can provide evidence for planning interventions to promote healthy and active ageing. Societal and lifestyle changes are sweeping through India, changing the living arrangements for the older population.^2 3^ Multigenerational households are giving way to nuclear families and small households.^2 3^ Under these changing circumstances, homes for the aged or residential care facilities are emerging and taking root rapidly.^2 3^

Fear of falling (FOF) is a term used to describe an individual’s loss of confidence in their ability to maintain balance, resulting in a worry about falling.^4^ The prevalence of FOF in the older population ranges from 29% to 92% with a history of falls and 12%–65% without a history of falls.^5^ In India, FOF is a common challenge affecting over one-third of the older population and affects their quality of life, physical well-being and social functioning.^6–8^ Moreover, it is associated with depression,^9^ cognitive decline and incidence of functional disability in the older population, it can also predict future falls.^10 11^ Several risk factors are associated with FOF, including visual impairment (VI), hearing impairment (HI), a history of falls, depression and physical functioning.^12 13^ A recent longitudinal study concluded that FOF is as detrimental as previous falls in limiting the daily activities among older people.^14^

Studies on active ageing, falls and FOF in older people are more common in high-income countries.^12 15^ In India, such studies are still at a nascent stage, with very few studies reporting on FOF and none reporting on the association of FOF between dual sensory impairment (DSI, combined vision and hearing impairment) and depression among the older people in residential care.^6–8^

The Hyderabad Ocular Morbidity in Elderly Study (HOMES) is a large study conducted among older residents (aged ≥60 years) living in homes for the aged in Hyderabad, Telangana, India.^16^ In addition to eye health examination, the study included an assessment of factors relevant to the health of older people, such as depression, falls, HI and FOF.^16^ Previous publications from this study reported on distance and near VI, dual sensory loss, depression, prevalence, and risk factors for falls in older people.^17–19^ In this paper, we report on the prevalence and associations of FOF with vision loss, hearing loss, dual sensory loss and depression among the older people living in homes for the aged facilities.

## Material and methods

### Sampling method and recruitment of the participants

The FOF was assessed as a part of a larger eye health and VI.^16^ A sample size of 916 participants was required based on an anticipated prevalence of 15% for avoidable VI, a 20% precision, a 25% non-response rate and a design effect of 1.4 to account for clustering for a cluster size of 40 people. Based on an anticipated prevalence of FOF of 33%, as reported by a previous study, the sample size required was 375 participants.^6^ Of the 76 centres, 46 were selected (including five homes for the pilot) based on the proximity to a referral centre for eye care services and the willingness of the homes to participate in the study. Participants aged ≥60 years at the time of enumeration residing in the home for the aged for at least one month were included in the study.

### Eye examination and other assessments

The examination procedures have been described in previous publications.^17–20^ Briefly, monocular visual acuity (VA) was assessed at a 3 m distance under ambient illumination using a logMAR ((logarithm of minimum angle of resolution) chart. The presenting and pinhole VAs were also assessed. Near vision was assessed using a logMAR chart at the standard distance of 40 cm. The anterior segment was examined using a portable slit lamp. A fundus examination and imaging were done for all participants and images were graded by trained graders. Mobility was classified as independently mobile and mobile with assistance based on the participants’ self-report and the interviewers’ observations, including using a walking stick or assisted by other people. The participants who were bedridden or in a wheelchair were not included in the study, though they were examined and provided with the necessary services.

### Non-clinical questionnaires

Trained investigators conducted detailed interviews with the participants using structured questionnaires.^16 20^ All interviews were conducted before the eye examination. These included assessing the personal and demographic information (age, education level, marital status and type of home) and systemic history (diabetes and hypertension). The Hindi Mini-Mental State Examination (HMSE) was used to assess cognitive status.^21^ All the participants with an HMSE score of 20 or more were further interviewed and assessed for depression using the Patient Health Questionnaire-9 (PHQ-9).^22^ The hearing was assessed using the Hearing Handicap Inventory for Elderly (HHIE-S) questionnaire, with a hearing aid, if any.^23^ Questionnaires were administered in the vernacular language (Telugu or Hindi).

FOF was assessed using the Short Falls Efficacy Scale (International) questionnaire.^24 25^ This questionnaire has seven questions on commonly performed tasks, including self-care, physical activity and participation in social events. The response grades included not at all concerned, somewhat concerned, fairly concerned and very concerned. All the response grades were added to get a cumulative FOF score.^24^ The history of previous falls was documented by asking, ‘Have you ever fallen on the floor in the last 1 year?’ in the vernacular language (Telugu or Hindi). The response was recorded as 0=no, 1=yes and 3=cannot remember.^18^ The participants were also asked if they had a fall in the last two weeks.

### Definitions

1. FOF: A cumulative score ≥9 was considered as an individual with FOF. It was used as a dichotomous variable to define FOF. It was also graded as low (≤8), moderate (9–13) and high concern (14–28).
2. VI: Presenting distance VA worse than 6/18 in the better eye, without HI.
3. HI: It was defined as an HHIE score <10 on the HHIE-S, without VI.
4. DSI: The presence of both VI and HI in an individual was considered DSI.
5. Depression: An individual with a cumulative score of ≥10 on the PHQ-9 was considered as having depression. It was graded as none/mild (0–9), moderate (10–14) and moderate to severe/severe (14–17).
6. Multimorbidity: It was defined as reporting two or more non-communicable diseases in an individual.
7. Polypharmacy: It was defined as the use of five or more medications by an individual on a daily basis.

### Patient and public involvement

The patients and the public were not involved in the design and conduct of the study.

### Data management

Data analysis was conducted using Stata V.14.0 (StataCorp LP, College Station, Tx).^26^ For descriptive statistics, continuous variables were analysed using the Student’s t-test, and the categorical variables were analysed using the χ^2^ test. The prevalence of FOF was estimated and presented with 95% CIs. In the multiple logistic regression analysis, FOF was used as a dichotomous outcome variable, and its associations with personal and sociodemographic variables (age, gender and education), body mass index, multimorbidity, polypharmacy, depression, HI, VI and DSI were evaluated. The variables were entered into the model one at a time, and the selection of covariates for the model was based on previous studies reporting on FOF.^27^ The Hosmer-Lemeshow goodness of fit test was used to assess the model fit. Variance inflation factors were used to test for collinearity between the covariates after fitting a multiple regression model. The adjusted ORs with 95% CIs were presented. Statistical significance was set at p<0.05 (two tailed), and the exact p-values were reported.

## Results

### Characteristics of the study participants

The personal and demographic characteristics of the participants have been described in previous publications.^19^ The HOMES study included 1182 participants; this included 271 participants who were bedridden and 98 participants with HMSE scores worse than 20.

Of the remaining 867 participants included in the analyses, 537 (61.6%) were women, 116 (13.4%) had no education, 518 (59.8%) reported hypertension and 263 (30.3%) reported diabetes. The mean (±SD) age of the participants was 74.2 (±8.3) years (range: 60–96 years). In terms of mobility status, 613 (70.7%) were independent, and the remaining 254 (29.3%) needed assistance from others or used walking aids for mobility. In total, 363 (41.9%) participants had multimorbidity and 161 (18.6%) reported polypharmacy.

### Sensory impairments

In total, 548 (63.2%) participants had no sensory loss, 134 (15.5%) had VI, 135 (15.6%) had HI and 50 (5.8%) had DSI. Among those with VI, the causes of VI were cataracts (44.0%; n=59), uncorrected refractive errors (35.5%; n=48), posterior capsular opacification (9%; n=12) and others (11%; n=15).

### Prevalence of FOF

The prevalence of FOF (moderate+high concern) was 56.1% (95% CI 52.7% to 59.4%; n=486). In total, 381 (43.9%) participants reported no concern about falling, 249 (28.7%) had a moderate concern and 237 (27.3%) reported a high concern. The prevalence of FOF varied with the nature of sensory loss, with the highest prevalence among those with DSI (92.0%), followed by HI (80.1%) and VI (57.5%). The prevalence of FOF among those with a history of falls was 69.4% (95% CI 63.2% to 75.2%) compared with 50.9% (95% CI 46.9% to 54.9%) among those without a history of falling. Those with multimorbidity had a significantly higher prevalence of FOF (table 1).

**Table 1:** Personal, demographic and health-related characteristics and prevalence of fear of falling (n=867) among the older population in homes for the aged centres

	Total in the sample	Fear of falling* n (row %)	Fear of falling — prevalence (95% CI)
Age group (years)			
60–69	263	118 (44.9)	9 (38.7 to 51.0)
70–79	345	186 (53.9)	53.9 (48.5 to 59.3)
80 and above	259	182 (70.3)	70.3 (64.3 to 75.8)
Gender			
Male	330	171 (51.8)	51.8 (46.3 to 57.3)
Female	537	315 (58.7)	58.7 (54.4 to 62.9))
Education level			
Any education	751	400 (53.3)	53.3 (49.6 to 56.9)
No education	116	86 (74.1)	74.1 (65.2 to 81.8)
Body mass index^†^			
Underweight/normal (≤24.9)	397	210 (52.9)	52.9 (47.8 to 57.9)
Overweight (25.0–29.9)	289	159 (55.0)	55.0 (49.1 to 60.8)
Obese (30 and above)	153	97 (63.4)	63.4 (55.2 to 71.0)
Hypertension			
No	349	157 (45.0)	45.0 (39.7 to 50.4)
Yes	518	329 (63.5)	63.5 (59.2 to 67.7)
Diabetes			
No	604	345 (57.1)	57.1 (53.1 to 61.1)
Yes	263	141 (53.6)	53.6 (47.4 to 59.7)
Mobility status			
Independently mobile	613	275 (44.9)	44.9 (40.9 to 49.9)
Mobility with support	254	211 (83.1)	83.1 (77.9 to 87.5)
Depression			
Mild/None	672	313 (46.6)	46.5 (42.7 to 50.4)
Moderate	104	89 (85.6)	85.6 (77.3 to 91.7)
Severe	91	84 (92.3)	92.3 (84.8 to 96.8)
History of fall in a year			
No fall	625	318 (50.9)	50.9 (46.9 to 54.9)
Falls reported	242	168 (69.4)	69.4 (63.2 to 75.2)
Vision impairment only			
No	733	409 (55.8)	55.8 (52.1 to 59.4)
Yes	134	77 (57.5)	57.4 (48.6 to 66.0)
Hearing impairment only			
No	732	377 (51.5)	51.5 (47.8 to 55.2)
Yes	135	109 (80.1)	80.1 (73.1 to 87.0)
Dual sensory impairment			
No	817	440 (53.9)	53.9 (50.4 to 57.3)
Yes	50	46 (92.0)	92.0 (80.8 to 97.8)
Multimorbidity			
No	504	261	51.7 (47.3 to 56.2)
Yes	363	225	62.0 (56.8 to 67.0)
Polypharmacy			
No	706	382	54.1 (50.3 to 57.8)
Yes	161	104	64.6 (56.7 to 72.0)
Total	867	486 (56.1)	56.1 (52.7 to 59.4)

*Fear of falling was defined as a score of ≥9 on the Short Falls Efficacy Scale (International) questionnaire.

†BMI data were not available on 28 participants.

BMI, body mass index.

**Figure 1:**
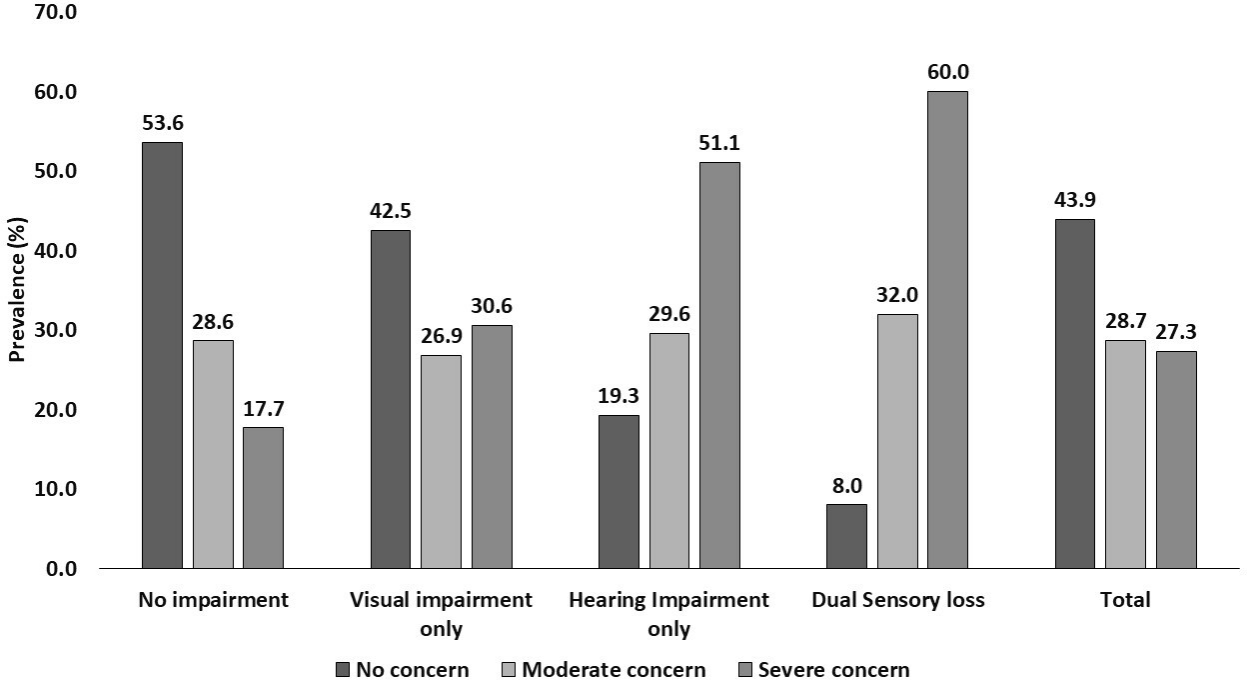
Sensory loss and fear of falling among the older people in residential care in India.

Figure 1 shows the grades of FOF among the participants without any impairment, HI, VI and DSI. The proportion of participants reporting a serious concern of falling was highest among those with DSI, followed by HI and VI (p<0.001).

### Risk factors for FOF

Table 2 shows the association between FOF and various risk factors. FOF was significantly associated with older age, high BMI and lack of education. The older participants with DSI had eleven times higher odds of FOF (OR 11.43; 95% CI 3.15 to 41.41, table 2) than those with no sensory impairment. HI was significantly associated with FOF (OR 3.34; 95% CI 1.96 to 5.72); however, VI (OR 1.51; 95% CI 0.95 to 2.40) was not significantly (p=0.08) associated. Those with moderate depression had seven times (OR 6.86; 95% CI 3.70 to 12.70) higher odds of FOF, and those with severe depression had eight times higher odds (OR 8.13; 95% CI 3.50 to 18.88). A history of falls over the past year, was also associated with an increase in odds for FOF (OR 1.53; 95% CI 1.03 to 2.26). Those needing assistance for mobility had five times higher odds of FOF (OR 4.84; 95% CI 3.1 to 7.44) compared with those with independent mobility. Polypharmacy and multimorbidity were not associated with FOF.

**Table 2:** Multiple logistic regression analysis, assessing the factors associated with fear of falling in older people living in residential care (n=839)

	Adjusted OR (95% CI)	P value
Age group (years)		
60–69	Reference	
70–79	1.27 (0.86 to 1.88)	0.233
80 and above	1.84 (1.16 to 2.92)	0.010
Gender		
Male	Reference	
Female	1.35 (0.95 to 1.94)	0.097
Education		
Any education	Reference	
No education	2.31 (1.34 to 3.99)	0.003
Body mass index		
Normal	Reference	
Overweight	1.28 (0.87 to 1.87)	0.215
Obese	2.03 (1.26 to 3.27)	0.003
Multimorbidity		
No	Reference	
Yes	1.22 (0.85 to 1.78)	0.273
Polypharmacy		
No	Reference	
Yes	1.36 (0.85 to 2.17)	0.193
Mobility status		
Independent mobility	Reference	
Mobility with support/aid	4.84 (3.15 to 7.44)	<0.001
Depression		
None	Reference	
Moderate	6.86 (3.71 to 12.70)	<0.001
Severe	8.13 (3.50 to 18.88)	<0.001
History of fall in last year		
No	Reference	
Yes	1.53 (1.03 to 2.26)	0.033
Dual sensory impairment		
No	Reference	
Yes	11.43 (3.15 to 41.42)	<0.001
Vision impairment only		
No	Reference	
Yes	1.51 (0.95 to 2.40)	0.084
Hearing impairment only		
No	Reference	
Yes	3.35 (1.96 to 5.72)	<0.001

## Discussion

Over half of the older people in residential care facilities had FOF. In addition, nearly 90% of those with combined HI and VI (DSI) reported FOF. Earlier studies done in India reported a lower prevalence of FOF compared with this study.^6–8^ The possible reasons for a higher prevalence in this study could be explained by the differences in the living conditions of the older residents. Earlier studies reported on the older population living in a community setting, whereas those in this study live in an institutional setting. Reports from other countries have shown a higher prevalence of FOF among residents in institutions.^28^ No previous studies have reported FOF among older residents in institutional care in India. One study reported a 42% prevalence of FOF among the older people in a hospital setting in India.^29^

Studies from other parts of the world have reported a higher prevalence of FOF among the older people living in institutional settings than those living in communities.^28 30 31^ However, the results from studies done in high-income countries cannot be extrapolated to India. Nursing homes and homes for the aged in high-income countries are well established, with clear protocols in terms of infrastructure requirements, dedicated personnel specialised in care for older people and laws governing the quality of care. Homes for the aged in India are relatively new, and there are no standard minimum requirements for establishing such institutions.^32^ The facilities, personnel and scope of service vary greatly based on the paying status of residents and the leadership of the facilities.^32^ These homes are largely are run by private and non-government entities.^32^ Due to these differences, the lifestyles of the older residents in homes in India and other high-income countries are not comparable.

The association between VI and FOF has been inconsistent across studies.^33–35^ Some have reported a positive association^36^ while others have found no associations.^35 37 38^ There was no association between VI and FOF in this study after adjusting for other covariates. A possible reason for the inconsistent associations across the studies is the differences in the definition used for VI. However, there could also be a small association, which most studies are underpowered to detect. We did find a positive association, but it was not statistically significant. It is also possible that the studies that reported an association between VI and FOF could have included those with DSI, which may have increased the association, as people with DSI were more likely to report FOF. Those with DSI had a substantially higher FOF compared with those with single sensory loss, either VI or HI. A recent study on the older population in nursing homes also reported a higher prevalence of FOF among those with DSI.^28^

The studies on eye health do not include the assessment of hearing as a routine procedure. We found that approximately 90% of those with DSI reported FOF, highlighting the need to assess both hearing and vision status in the older population for comphehensive care and cross referral. It is also useful to assess DSI when carrying out research studies in older individuals. Finally, though earlier studies reported a higher prevalence of FOF among women, there was no significant association with gender in this study.^39^ FOF is associated independently with VI and HI.^34^ A recent study reported a higher prevalence of FOF among nursing home residents with sensory loss, as over half of them had FOF.^28^ A study by Murphy *et al* reported that older people who had restricted their daily activities out of FOF had a higher burden of depressive symptoms than those who only reported FOF.^40^ A meta-analysis reported that untreated depression is significantly associated with an increased risk of falling, which in turn may lead to FOF.^27^

FOF was associated with depression in this study, which is consistent with other studies.^13 30 36 41–46^ Causality is impossible to demonstrate using a cross sectional study design; however, several pathways for FOF leading to depression could be hypothesised. FOF is associated with reduced physical activity in older people.^47^ This is evident in the current study where a higher odds for FOF among those needing assistance for mobility is noted. The reduced activity results in a more sedentary lifestyle, increasing social isolation and leading to depression. However, it is also possible that those with higher levels of depression tend to become sedentary and limit their social interaction and mobility.

In our previous publication, we reported that the odds of having depression is ten times higher in people with DSI than those with a single sensory loss.^19^ In this study, HI was independently associated with FOF after adjusting for other covariates. The possible explanation for this finding could be the changes in gait balance due to HI, leading to an increased FOF. A greater FOF was reported among those with lower gait velocity.^48^

Independent mobility is a measure of physical function, and a loss of it is associated with falls and FOF.^49–51^ Poor physical function may result in a loss of confidence among older people, resulting in an increased FOF.^50^ In a longitudinal study, FOF was associated with a lack of independent mobility and poor physical performance at the end of two years.^14^ This indicates the need to address FOF in the older population to prevent a decline in physical functions.

The association between falls and FOF is reported in a few studies in India.^7 37^ FOF was higher among those with a history of falls in India.^7 33^ Adverse consequences of falls in the older people are also well known, which in itself may lead to FOF. Fear is an innate response that has an evolutionary importance and is a protective instinct. Similarly, FOF could act as an instinctual defence mechanism to prevent falls but only to a certain threshold.^31 52^ Beyond this threshold, FOF could impair physical functioning and negatively impact the quality of life in older people.^31 52^ A recent systematic review has concluded that FOF adversely impacts the quality of life of older residents in community settings and institutional care.^53^

Several interventions have been reported to address FOF in older people living in communities and nursing homes. These interventions include cognitive and behavioural interventions, occupational therapy and physical exercises, including balance training, yoga and tai chi.^9 43 54–59^ In addition to addressing FOF, these interventions may also have a resonating effect, alleviating depression, leading to better quality of life and promoting well-being in the older population.^60–62^ However, studies on the impact of such interventions that address FOF are limited in India. The older residents living in institutional care present a unique opportunity where interventions can be studied systematically and applied to more individuals in one setting, compared to older population diffused across communities. Studies also indicate an association between FOF and cognitive decline,^36 63^ which underpins the need to address FOF among the older people.

This study benefits from a large cohort of older participants and a comprehensive assessment (inclusion of DSI and depression). Some limitations include our decision to exclude participants with severe mobility issues and those with cognitive impairment, as indicated by the lower HSME scores. This may have led to an underestimation of the prevalence of FOF in our study. However, it is unlikely to have led to an inaccurate assessment of associations. Environmental and infrastructure factors also influence FOF, which were not evaluated in our study. A direct extrapolation of our results to those who live in the communities is limited due to the differences in the types of residences and the activities of people. Moreover, while vision assessment was done using the standard procedures used in the clinic, hearing was assessed using a questionnaire, which is not an objective assessment. This limitation could have led to an underestimation of the prevalence of HI in our study. HOMES was an observational study hence causality cannot be established.

In conclusion, FOF is common among the older population in residential care in India. It is especially common among those with depression and DSI. With the increase in the older population and the rapid increase in the number of homes for the aged in urban areas, these findings are relevant for policy and healthcare service planning for older people. The findings of this study can help inform planners to develop appropriate interventions to address FOF in the older population and promote healthy and active ageing among these residents.

## Supplementary Material

Reviewer comments

Author's manuscript

## Data Availability

No data are available.

## References

[1] UN . World population prospects 2017 United Nations, Department of economic and social affairs, population division 2017. 2017.

[2] Agrawal S . Effect of living arrangements on health status of elderly in India. Asian Populat Stud 2012;8:87–101. 10.1080/17441730.2012.646842 PMC5575814 28868080

[3] B Mane A . Ageing in India: some social challenges to elderly care. J Gerontol Geriat Res 2016;05:e136. 10.4172/2167-7182.1000e136

[4] Legters K . Fear of falling. Phys Ther 2002;82:264–72. 11869155

[5] Malini FM , Lourenço RA , Lopes CS . Prevalence of fear of falling in older adults, and its associations with clinical, functional and Psychosocial factors: the frailty in Brazilian older people-Rio de Janeiro study. Geriatr Gerontol Int 2016;16:336–44. 10.1111/ggi.12477 25869919

[6] Mane AB , Sanjana T , Patil PR , et al . Prevalence and correlates of fear of falling among elderly population in urban area of Karnataka, India. J Midlife Health 2014;5:150–5. 10.4103/0976-7800.141224 25317002 PMC4195189

[7] Pitchai P , Dedhia HB , Bhandari N , et al . Prevalence, risk factors, circumstances for falls and level of functional independence among geriatric population - A descriptive study. Indian J Public Health 2019;63:21–6. 10.4103/ijph.IJPH_332_17 30880733

[8] Dhavale S , Singh S , Parasher RK . Factors associated with fear of falls in Indian elderly: A systematic review. Ind Jour of Publ Health Rese & Develop 2019;10:345. 10.5958/0976-5506.2019.02825.0

[9] Dukyoo J , Juhee L , Lee S-M . A meta-analysis of fear of falling treatment programs for the elderly. West J Nurs Res 2009;31:6–16. 10.1177/0193945908320466 18667626

[10] Shirooka H , Nishiguchi S , Fukutani N , et al . Cognitive impairment is associated with the absence of fear of falling in community-dwelling frail older adults. Geriatr Gerontol Int 2017;17:232–8. 10.1111/ggi.12702 26792588

[11] Auais M , French S , Alvarado B , et al . Fear of falling predicts incidence of functional disability 2 years later: A perspective from an international cohort study. J Gerontol A Biol Sci Med Sci 2018;73:1212–5. 10.1093/gerona/glx237 29220420 PMC6093362

[12] Jung D . Fear of falling in older adults: comprehensive review. Asian Nurs Res (Korean Soc Nurs Sci) 2008;2:214–22. 10.1016/S1976-1317(09)60003-7 25029959

[13] Mishra N , Mishra AK , Bidija M . A study on correlation between depression, fear of fall and quality of life in elderly individuals. Int J Res Med Sci 2017;5:1456. 10.18203/2320-6012.ijrms20171245

[14] Liu M , Hou T , Li Y , et al . Fear of falling is as important as multiple previous falls in terms of limiting daily activities: a longitudinal study. BMC Geriatr 2021;21:350. 10.1186/s12877-021-02305-8 34098904 PMC8185919

[15] Scheffer AC , Schuurmans MJ , van Dijk N , et al . Fear of falling: measurement strategy, prevalence, risk factors and consequences among older persons. Age Ageing 2008;37:19–24. 10.1093/ageing/afm169 18194967

[16] Marmamula S , Barrenkala NR , Challa R , et al . Hyderabad ocular morbidity in elderly study (HOMES) - rationale, study design and methodology. Ophthalmic Epidemiol 2020;27:83–92. 10.1080/09286586.2019.1683867 31658840 PMC6961304

[17] Marmamula S , Barrenakala NR , Challa R , et al . Prevalence and risk factors for visual impairment among elderly residents in 'homes for the aged' in India: the Hyderabad ocular morbidity in elderly study (HOMES). Br J Ophthalmol 2021;105:32–6. 10.1136/bjophthalmol-2019-315678 32217544 PMC7116480

[18] Marmamula S , Barrenkala NR , Challa R , et al . Falls and visual impairment among elderly residents in 'homes for the aged' in India. Sci Rep 2020;10:13389. 10.1038/s41598-020-70066-2 32770042 PMC7414840

[19] Marmamula S , Kumbham TR , Modepalli SB , et al . Depression, combined visual and hearing impairment (dual sensory impairment): a hidden multi-morbidity among the elderly in residential care in India. Sci Rep 2021;11:16189. 10.1038/s41598-021-95576-5 34376737 PMC8355224

[20] Marmamula S , Barrenkala NR , Challa R , et al . Hyderabad ocular morbidity in elderly study (HOMES) – rationale, study design and methodology. Ophthalmic Epidemiol 2020;27:83–92. 10.1080/09286586.2019.1683867 31658840 PMC6961304

[21] Folstein MF , Folstein SE , McHugh PR . Mini-mental state". A practical method for grading the cognitive state of patients for the clinician. J Psychiatr Res 1975;12:189–98. 10.1016/0022-3956(75)90026-6 1202204

[22] Kroenke K , Spitzer RL , Williams JBW , et al . The patient health questionnaire somatic, anxiety, and depressive symptom scales: a systematic review. Gen Hosp Psychiatry 2010;32:345–59. 10.1016/j.genhosppsych.2010.03.006 20633738

[23] Ventry IM , Weinstein BE . The hearing handicap inventory for the elderly: a new tool. Ear Hear 1982;3:128–34. 10.1097/00003446-198205000-00006 7095321

[24] Kempen GIJM , Yardley L , van Haastregt JCM , et al . The short FES-I: a shortened version of the falls efficacy scale-International to assess fear of falling. Age Ageing 2008;37:45–50. 10.1093/ageing/afm157 18032400

[25] Tinetti ME , Richman D , Powell L . Falls efficacy as a measure of fear of falling. J Gerontol 1990;45:239–43. 10.1093/geronj/45.6.p239 2229948

[26] StataCorp . Stata Statistical Software: Release 14. College Station, TX: StataCorp LP, 2014.

[27] Deandrea S , Lucenteforte E , Bravi F , et al . Risk factors for falls in community-dwelling older people: a systematic review and meta-analysis. Epidemiology 2010;21:658–68. 10.1097/EDE.0b013e3181e89905 20585256

[28] Lach HW , Lozano AJ , Hanlon AL , et al . Fear of falling in sensory impaired nursing home residents. Aging Ment Health 2020;24:474–80. 10.1080/13607863.2018.1537359 30621452 PMC6616018

[29] Dhar M , Kaeley N , Mahala P , et al . The prevalence and associated risk factors of fear of fall in the elderly: A hospital-based, cross-sectional study. Cureus 2022;14:e23479. 10.7759/cureus.23479 35475069 PMC9035266

[30] Chou K-L , Yeung FKC , Wong ECH . Fear of falling and depressive symptoms in Chinese elderly living in nursing homes: fall efficacy and activity level as mediator or moderator. Aging Mental Health 2005;9:255–61. 10.1080/13607860500114035 16019279

[31] Gillespie SM , Friedman SM . Fear of falling in New long-term care enrollees. J Am Med Dir Assoc 2007;8:307–13. 10.1016/j.jamda.2007.04.006 17570309 PMC2043160

[32] Liebig PS . Old-age homes and services: old and new approaches to aged care. J Aging Soc Policy 2003;15:159–78. 10.1300/J031v15n02_10 14696695

[33] Subramanian MS , Singh V , Chatterjee P , et al . Prevalence and predictors of falls in a health-seeking older population: an outpatient-based study. Aging Med (Milton) 2020;3:25–31. 10.1002/agm2.12096 32232189 PMC7099749

[34] White UE , Black AA , Wood JM , et al . Fear of falling in vision impairment. Optom Vis Sci 2015;92:730–5. 10.1097/OPX.0000000000000596 25930978

[35] Donoghue OA , Ryan H , Duggan E , et al . Relationship between fear of falling and mobility varies with visual function among older adults. Geriatr Gerontol Int 2014;14:827–36. 10.1111/ggi.12174 24215140

[36] Vo THM , Nakamura K , Seino K , et al . Fear of falling and cognitive impairment in elderly with different social support levels: findings from a community survey in central Vietnam. BMC Geriatr 2020;20:141. 10.1186/s12877-020-01533-8 32299392 PMC7164140

[37] Friedman SM , Munoz B , West SK , et al . Falls and fear of falling: which comes first? A longitudinal prediction model suggests strategies for primary and secondary prevention. J Am Geriatr Soc 2002;50:1329–35. 10.1046/j.1532-5415.2002.50352.x 12164987

[38] Deshpande N , Metter EJ , Lauretani F , et al . Activity restriction induced by fear of falling and objective and subjective measures of physical function: a prospective cohort study. J Am Geriatr Soc 2008;56:615–20. 10.1111/j.1532-5415.2007.01639.x 18312314 PMC2645621

[39] Gazibara T , Kurtagic I , Kisic-Tepavcevic D , et al . Falls, risk factors and fear of falling among persons older than 65 years of age. Psychogeriatrics 2017;17:215–23. 10.1111/psyg.12217 28130862

[40] Murphy SL , Williams CS , Gill TM . Characteristics associated with fear of falling and activity restriction in community-living older persons. J Am Geriatr Soc 2002;50:516–20. 10.1046/j.1532-5415.2002.50119.x 11943049 PMC3046411

[41] van Haastregt JCM , Zijlstra GAR , van Rossum E , et al . Feelings of anxiety and symptoms of depression in community-living older persons who avoid activity for fear of falling. Am J Geriatr Psychiatry 2008;16:186–93. 10.1097/JGP.0b013e3181591c1e 18310549

[42] Cumming RG , Salkeld G , Thomas M , et al . Prospective study of the impact of fear of falling on activities of daily living, SF-36 scores, and nursing home admission. J Gerontol A Biol Sci Med Sci 2000;55:M299–305. 10.1093/gerona/55.5.m299 10819321

[43] Huang TT , Chung ML , Chen FR , et al . Evaluation of a combined cognitive-behavioural and exercise intervention to manage fear of falling among elderly residents in nursing homes. Aging Ment Health 2016;20:2–12. 10.1080/13607863.2015.1020411 25791743

[44] Berkman LF , Sekher T , Capistrant B , et al . Social networks, family, and care giving among older adults in India. aging in Asia: findings from new and emerging data initiatives: national academies press (US). 2012. 23077756

[45] Singh L , Singh PK , Arokiasamy P . Social network and mental health among older adults in rural Uttar Pradesh, India: A cross-sectional study. J Cross Cult Gerontol 2016;31:173–92. 10.1007/s10823-016-9286-0 26879450

[46] Hajek A , Bock J-O , König H-H . Psychological correlates of fear of falling: findings from the German aging survey. Geriatr Gerontol Int 2018;18:396–406. 10.1111/ggi.13190 29143433

[47] Dsouza SA , Rajashekar B , Dsouza H , et al . Falls in Indian older adults: a barrier to active ageing. Asian J Gerontol Geriatr 2014;9:1–8.

[48] Reelick MF , van Iersel MB , Kessels RPC , et al . The influence of fear of falling on gait and balance in older people. Age Ageing 2009;38:435–40. 10.1093/ageing/afp066 19451658

[49] Ayoubi F , Launay C , Annweiler C , et al . Fear of falling, falls, and gait variability in older community-dwelling individuals: is there an association J Am Geriatr Soc 2013;61:1236–8. 10.1111/jgs.12350 23855862

[50] Brouwer B , Musselman K , Culham E . Physical function and health status among seniors with and without a fear of falling. Gerontology 2004;50:135–41. 10.1159/000076771 15114034

[51] Jefferis BJ , Iliffe S , Kendrick D , et al . How are falls and fear of falling associated with objectively measured physical activity in a cohort of community-dwelling older men BMC Geriatr 2014;14:114. 10.1186/1471-2318-14-114 25348492 PMC4223846

[52] Murphy SL , Dubin JA , Gill TM . The development of fear of falling among community-living older women: predisposing factors and subsequent fall events. J Gerontol A Biol Sci Med Sci 2003;58:M943–7. 10.1093/gerona/58.10.m943 14570863 PMC3050034

[53] Schoene D , Heller C , Aung YN , et al . A systematic review on the influence of fear of falling on quality of life in older people: is there a role for falls Clin Interv Aging 2019;14:701–19. 10.2147/CIA.S197857 31190764 PMC6514257

[54] Tennstedt S , Howland J , Lachman M , et al . A randomized, controlled trial of a group intervention to reduce fear of falling and associated activity restriction in older adults. J Gerontol B Psychol Sci Soc Sci 1998;53:384–92. 10.1093/geronb/53b.6.p384 9826971

[55] Walker JE , Howland J . Falls and fear of falling among elderly persons living in the community: occupational therapy interventions. Am J Occup Ther 1991;45:119–22. 10.5014/ajot.45.2.119 2035588

[56] Zhang J-G , Ishikawa-Takata K , Yamazaki H , et al . The effects of Tai Chi Chuan on physiological function and fear of falling in the less robust elderly: an intervention study for preventing falls. Arch Gerontol Geriatr 2006;42:107–16. 10.1016/j.archger.2005.06.007 16125805

[57] Sattin RW , Easley KA , Wolf SL , et al . Reduction in fear of falling through intense Tai Chi exercise training in older, Transitionally frail adults. J Am Geriatr Soc 2005;53:1168–78. 10.1111/j.1532-5415.2005.53375.x 16108935

[58] Liu TW , Ng GYF , Ng SSM . Effectiveness of a combination of cognitive behavioral therapy and task-oriented balance training in reducing the fear of falling in patients with chronic stroke: study protocol for a randomized controlled trial. Trials 2018;19:168. 10.1186/s13063-018-2549-z 29514677 PMC5842580

[59] Sjösten N , Vaapio S , Kivelä S-L . The effects of fall prevention trials on depressive symptoms and fear of falling among the aged: a systematic review. Aging Ment Health 2008;12:30–46. 10.1080/13607860701366079 18297477

[60] Chobe S , Chobe M , Metri K , et al . Impact of yoga on cognition and mental health among elderly: a systematic review. Complement Ther Med 2020;52. 10.1016/j.ctim.2020.102421 32951703

[61] Cramer H , Lauche R , Langhorst J , et al . Yoga for depression: a systematic review and meta-analysis. Depress Anxiety 2013;30:1068–83. 10.1002/da.22166 23922209

[62] Zettergren KK , Lubeski JM , Viverito JM . Effects of a yoga program on postural control, mobility, and gait speed in community-living older adults: a pilot study. J Geriatr Phys Ther 2011;34:88–94. 10.1519/JPT.0b013e31820aab53 21937898

[63] Noh H-M , Roh YK , Song HJ , et al . Severe fear of falling is associated with cognitive decline in older adults: a 3-year prospective study. J Am Med Dir Assoc 2019;20:1540–7. 10.1016/j.jamda.2019.06.008 31351857

