# Supplementary material for: Cross-sectional study of prevalence and correlates of fear of falling in the older people in residential care in India: the Hyderabad Ocular Morbidity in Elderly Study (HOMES)
Source: BMJ Open. 2024 May 28;14(5):e080973. doi: 10.1136/bmjopen-2023-080973 (PMC11138276; doi:10.1136/bmjopen-2023-080973)
Supplement: Author's manuscript [file bmjopen-2023-080973.draft_revisions.pdf]

BMJ Open is committed to open peer review. As part of this commitment we make the peer review history of every article we publish publicly available.

When an article is published we post the peer reviewers' comments and the authors' responses online. We also post the versions of the paper that were used during peer review. These are the versions that the peer review comments apply to.

The versions of the paper that follow are the versions that were submitted during the peer review process. They are not the versions of record or the final published versions. They should not be cited or distributed as the published version of this manuscript.

BMJ Open is an open access journal and the full, final, typeset and author-corrected version of record of the manuscript is available on our site with no access controls, subscription charges or pay-per-view fees (http://bmjopen.bmj.com).

If you have any questions on BMJ Open's open peer review process please email info.bmjopen@bmj.com

A cross sectional study of prevalence and correlates of fear of falling in the elderly in residential care in India – the Hyderabad Ocular Morbidity in Elderly Study

Journal:	*BMJ Open*
Manuscript ID	bmjopen-2023-080973
Article Type:	Original research
Date Submitted by the Author:	15-Oct-2023

Complete List of Authors:	Marmamula, Srinivas; LV Prasad Eye Institute, GPR ICARE; University of New South Wales<br>Kumbham, Thirupathi Reddy; LV Prasad Eye Institute, GPR ICARE<br>Modepalli, Satya Brahmanandam ; LV Prasad Eye Institute, Gullapalli Pratibha Rao International Centre for Advancement of Rural Eye care<br>Barrenkala, Navya Rekha; LV Prasad Eye Institute<br>Keeffe, Jill; LV Prasad Eye Institute, GPR ICARE<br>Friedman, David; Massachusetts Eye and Ear Infirmary

Keywords:	Aging, EPIDEMIOLOGY, GERIATRIC MEDICINE, PUBLIC HEALTH

SCHOLARONE™
Manuscripts

I, the Submitting Author has the right to grant and does grant on behalf of all authors of the Work (as defined in the below author licence), an exclusive licence and/or a non-exclusive licence for contributions from authors who are: i) UK Crown employees; ii) where BMJ has agreed a CC-BY licence shall apply, and/or iii) in accordance with the terms applicable for US Federal Government officers or employees acting as part of their official duties; on a worldwide, perpetual, irrevocable, royalty-free basis to BMJ Publishing Group Ltd ("BMJ") its licensees and where the relevant Journal is co-owned by BMJ to the co-owners of the Journal, to publish the Work in this journal and any other BMJ products and to exploit all rights, as set out in our licence.

The Submitting Author accepts and understands that any supply made under these terms is made by BMJ to the Submitting Author unless you are acting as an employee on behalf of your employer or a postgraduate student of an affiliated institution which is paying any applicable article publishing charge ("APC") for Open Access articles. Where the Submitting Author wishes to make the Work available on an Open Access basis (and intends to pay the relevant APC), the terms of reuse of such Open Access shall be governed by a Creative Commons licence – details of these licences and which Creative Commons licence will apply to this Work are set out in our licence referred to above.

Other than as permitted in any relevant BMJ Author's Self Archiving Policies, I confirm this Work has not been accepted for publication elsewhere, is not being considered for publication elsewhere and does not duplicate material already published. I confirm all authors consent to publication of this Work and authorise the granting of this licence.

For peer review only

A cross sectional study of prevalence and correlates of fear of falling in the elderly in residential care in India – the Hyderabad Ocular Morbidity in Elderly Study

Srinivas Marmamula,[1,2,3,4,5*] Thirupathi Reddy K,[1,2] Satya Brahmanandam Modepalli, [1,2]

Navya Rekha Barrenkala,[1] Jill E Keeffe,[1] David S Friedman[6]

[1] Allen Foster Community Eye Health Research Centre, Gullapalli Pratibha Rao International Centre for Advancement of Rural Eye care, L V Prasad Eye Institute, Hyderabad, India

[2] Brien Holden Institute of Optometry and Vision Science, L V Prasad Eye Institute, Hyderabad, India

[3] Wellcome Trust / Department of Biotechnology India Alliance, L V Prasad Eye Institute, Hyderabad, India

[4] School of Optometry and Vision Science, University of New South Wales, Sydney, Australia

[5] Harvard T.H. Chan School of Public Health, Boston, MA, USA

[6] Massachusetts Eye and Ear, Harvard Medical School, Harvard Medical School, Department of Ophthalmology, Boston, USA

Running Title: Fear of falling in the elderly people in India

***Corresponding author:** Dr. Srinivas Marmamula, Gullapalli Pratibha Rao International Centre for Advancement of Rural Eye care, L V Prasad Eye Institute, Hyderabad, India. 500034. Email: sri.marmamula@lvpei.org

Total number of words: 3217 words excluding title page, abstract, acknowledgments, references, tables and figure legends

For peer review only - http://bmjopen.bmj.com/site/about/guidelines.xhtml

ABSTRACT

Objective: To report the prevalence and risk factors for the fear of falling (FOF) among elderly individuals living in residential care facilities in India.

Design: Cross sectional study

Setting: Homes for the aged centres in Hyderabad, India

Participants: The study included individuals aged ≥60 years from homes for the aged centres. The participants underwent a comprehensive eye examination in make-shift clinics in homes. Trained investigators collected the personal and demographic information of the participants using the Patient Health Questionnaire-9 and Hearing Handicap Inventory for Elderly questionnaire in the vernacular language. FOF was assessed using the Short Falls Efficacy Scale. The presence of hearing and vision impairment in the same individual was considered dual sensory impairment (DSI). A multiple logistic regression analysis was done to assess the factors associated with FOF.

Primary Outcome measure: Fear of Falling (FOF)

Results: In total, 867 participants were included from 41 homes for the aged centres in the analyses. The mean (±SD) age of the participants was 74.2 (±8.3) years (range: 60–96 years). The prevalence of FOF was 56.1% (95% CI:52.7–59.4; n=486. The multivariate analysis showed that those with DSI were eleven times more likely to report FOF than those with no impairment (OR:11.14; 95% CI:3.15–41.4.) Similarly, those with moderate depression were seven times more likely (OR:6.85; 95% CI:3.70–12.70), and those with severe depression were eight times (OR:8.13; 95% CI: 3.50–18.90) more likely to report FOF. A history of falls in the last year was also associated with increased odds for FOF (OR:1.52; 95% CI:1.03–2.26).

Conclusion: FOF is common among the elderly individuals in residential care in India. Depression, falling in the previous year, and DSI were strongly associated with FOF. A cross disciplinary approach may be required to address FOF in the elderly in residential care in India.

Keywords: Fear of falling, dual sensory impairment, depression, older people

For peer review only

For peer review only - http://bmjopen.bmj.com/site/about/guidelines.xhtml

ARTICLE SUMMARY:

Strengths and limitations of the study

- A cross sectional study that included a large number of elderly in residential care where comprehensive assessment was conducted
- Provided insights on prevalence and various risk factors associated with fear of falling.
- Hearing assessment was based on questionnaire and could have resulted in underestimating of prevalence of lower grades of hearing impairments.
- The results are generalizable only to the elderly in residential care settings.

For peer review only

For peer review only - http://bmjopen.bmj.com/site/about/guidelines.xhtml

The proportion of the elderly among India's population is on the rise, and it is expected to reach 20% of the total population by 2050.[1] This demographic shift will dramatically change the nature of healthcare in India, and research on healthy ageing among the elderly population is needed. Such research can provide evidence for planning interventions to promote healthy and active ageing. Societal and lifestyle changes are sweeping through India, changing the living arrangements for the elderly population.[2 3] Multi-generational households are giving way to nuclear families and small households.[2 3] Under these changing circumstances, homes for the aged or residential care facilities are emerging and taking root rapidly.[2 3]

Fear of falling (FOF) is a term used to describe an individual's loss of confidence in their ability to maintain balance, resulting in a worry about falling.[4] The prevalence of FOF in the elderly population ranges from 29–92% with a history of falls and 12–65% without a history of falls.[5] In India, FOF is a common challenge affecting over a third of the elderly population[6-8] and affects their quality of life, physical well-being, and social functioning. Moreover, it is associated with depression,[9] cognitive decline, and incidence of functional disability in the elderly population and can predict future falls.[10 11] Several risk factors are associated with FOF, including vision impairment, hearing impairment, a history of falls, depression, and physical functioning.[12 13] A recent longitudinal study concluded that FOF is as detrimental as previous falls in limiting the daily activities among the elderly.[14]

Studies on active ageing, falls, and FOF among the elderly are more common in high-income countries.[12 15] In India, such studies are still in a nascent stage, with very few studies reporting on FOF [6-8] and none reporting on the association of FOF with Dual Sensory Impairment (DSI, combined vision and hearing impairment) and depression among the elderly population in residential care.

The Hyderabad Ocular Morbidity in Elderly Study (HOMES) is a large study conducted among elderly residents (aged $\geq$ 60 years) living in homes for the aged in Hyderabad, Telangana, India.[16] In addition to eye health, the study included an assessment of factors relevant to the health of the elderly residents, such as depression, falls, hearing impairment, and FOF.[16] Previous publications from this study reported on distance and near vision impairment, dual sensory loss, depression, prevalence, and risk factors for falls in the

For peer review only - http://bmjopen.bmj.com/site/about/guidelines.xhtml

elderly.[17-19] In this paper, we report on the prevalence and associations of FOF with vision loss, hearing loss, dual sensory loss, and depression among the elderly living in homes for the aged in India.

MATERIAL AND METHODS

Ethics approval

The protocol for the HOMES study was approved by the Institutional Review Board of the Hyderabad Eye Research Foundation, L V Prasad Eye Institute, Hyderabad. The study was performed in accordance with the Declaration of Helsinki. Each participant provided written informed consent for their participation in the study. The data collection for this study was carried out during 2017-2019.

Sampling method and recruitment of the participants

The FOF was assessed as a part of a larger eye health and visual impairment study.[16] A sample size of 916 participants was required based on an anticipated prevalence of 15% for avoidable visual impairment, a 20% precision, a 25% non-response rate, and a design effect of 1.4 to account for clustering for a cluster size of 40 people. Based on anticipated prevalence of FOF of 33% as reported by a previous study, the sample required was 375 participants.[6] Of the 76 centres, 46 were selected (including five homes for the pilot) based on the proximity to a referral centre for eye care services and the willingness of the homes to participate in the study. Participants aged ≥60 years at the time of enumeration residing in the home for the aged for at least one month were included in the study.

Eye examination and other assessments

The examination procedures have been described in previous publications.[17-20] Briefly, monocular visual acuity (VA) was assessed at a three-meter distance under ambient illumination using a logMAR chart. The presenting and pinhole visual acuities were also assessed. Near vision was assessed using a logMAR chart at the standard distance of 40 cm. The anterior segment was examined using a portable slit lamp. A fundus examination and imaging were done for all participants and were graded by trained graders. Mobility was classified as independently mobile and mobile with assistance based on the participants' self-report and the interviewers' observations, including using a walking stick or assisted by other people. The participants who were bedridden or in a wheelchair were not included in the study, though they were examined and provided with the necessary services.

For peer review only - http://bmjopen.bmj.com/site/about/guidelines.xhtml

Non-clinical questionnaires

Trained investigators conducted detailed interviews with the participants using structured questionnaires.[16 20] All interviews were conducted before the eye examination. These included assessing the personal and demographic information (age, education level, marital status, and type of home) and systemic history (diabetes and hypertension). The Hindi Mini-Mental State Examination (HMSE) was used to assess cognitive status.[21] All the participants with an HMSE score of 20 or more were further interviewed and assessed for depression using the Patient Health Questionnaire-9 (PHQ-9).[22] The hearing was assessed using the Hearing Handicap Inventory for Elderly (HHIE-S) questionnaire with a hearing aid, if any.[23] Questionnaires were administered in the vernacular languages (Telugu or Hindi).

Fear of Falling (FOF) was assessed using the Short Falls Efficacy Scale (SFES - International) questionnaire.[24 25] This questionnaire has seven questions on commonly performed tasks, including self-care, physical activity, and participation in social events. The response grades included Not at All Concerned, Somewhat Concerned, Fairly Concerned, and Very Concerned. All the response grades were added to get a cumulative FOF score.[24] The history of previous falls was documented by asking, "Have you ever fallen on the floor in the last one year?" in the vernacular language (Telugu or Hindi). The response was recorded as 0=No, 1=Yes, and 3= Cannot remember.[18] Moreover, the participants were also asked if they had a fall in the last two weeks.

Definitions

a. Fear of Falling (FOF): A cumulative score ≥9 was considered as an individual with FOF. It was used as a dichotomous variable to define FOF. It was also graded as low (≤8), moderate (9-13), and high concern (14-28).
b. Vision Impairment (VI): Presenting distance visual acuity (VA) worse than 6/18 in the better eye, without hearing impairment (HI).
c. Hearing impairment (HI): It was defined as an HHIE Score <10 on the HHIE-S, without visual impairment (VI).
d. Dual Sensory Impairment (DSI): The presence of both VI and HI in an individual was considered DSI.
e. Depression: An individual with a cumulative score of ≥10 on the PHQ-9 was considered as having depression. It was graded as none/mild (0-9), moderate (10-14), and moderate-severe/severe (14-17).

For peer review only - http://bmjopen.bmj.com/site/about/guidelines.xhtml

f. Multimorbidity: It was defined as reporting two or more non-communicable diseases (NCDs).
g. Polypharmacy: It was defined as the use of five or more medications on a daily basis.

Data management

Data analysis was conducted using Stata 14.0 (Stata Corp LP, College Station, TX).[26] For descriptive statistics, continuous variables were analysed using the Student's t-test, and the categorical variables were analysed using the chi-square test. The prevalence of FOF was estimated and presented with 95% confidence intervals (CI). In the multiple logistic regression analysis, FOF was used as a dichotomous outcome variable, and its association with personal and sociodemographic variables (age, gender, education), systemic conditions (self-report of hypertension and diabetes), depression, HI, VI, and DSI was evaluated. The variables were entered into the model one at a time, and the selection of covariates for the model was based on previous studies reporting on FOF.[27] The Hosmer-Lemeshow goodness of fit test was used to assess the model fit. Variance inflation factors (VIF) were used to test for collinearity between the covariates after fitting a multiple regression model. The adjusted odds ratio (OR) with 95% confidence intervals (CI) was presented. Statistical significance was set at $p<0.05$ (two-tailed), and the exact p-values were reported.

RESULTS

Characteristics of the study participants

The personal and demographic characteristics of the participants have been described in previous publications.[19] Among the 867 participants included in the analyses, 537 (61.6%) were women, 116 (13.4%) had no education, 518 (59.8%) reported hypertension, and 263 (30.3%) reported diabetes. The mean (±SD) age of the participants was 74.2 (±8.3) years (range: 60–96 years). In terms of mobility status, 613 (70.7%) were independent, and the remaining 254 (29.3%) needed assistance from others or used walking aids for mobility. In total, 363 (41.9%) participants had multimorbidity and 161 (18.6%) reported polypharmacy.

Sensory impairments

In total, 548 (63.2%) participants had no sensory loss, 134 (15.5%) had VI, 135 (15.6%) had HI, and 50 (5.8%) had DSI. Among those with VI, the cause of VI was cataract (44.0%; n=59), uncorrected refractive errors (35.5%; n=48), posterior capsular opacification (9%; n=12), and others (11%; n=15).

For peer review only - http://bmjopen.bmj.com/site/about/guidelines.xhtml

Prevalence of FOF

The prevalence of FOF (moderate + high concern) was 56.1% (95% CI: 52.7–59.4; n=486). In total, 381 (43.9%) participants reported no concern about falling, 249 (28.7%) had a moderate concern, and 237 (27.3%) reported a high concern. The prevalence of FOF varied with the nature of sensory loss, with the highest prevalence among those with DSI (92.0%), followed by HI (80.1%) and VI (57.5%). The prevalence of FOF among those with a history of falls was 69.4% (95% CI: 63.2–75.2) compared to 50.9% (95% CI: 46.9–54.9) among those without a history of falling. Those with multimorbidity had a significantly higher prevalence of FOF (Table 1).

Table 1: Personal, demographic, and health-related characteristics and prevalence of fear of falling (n=867).

	Total in the sample	Fear of Falling † n (row %)	Fear of Falling - Prevalence (95% CI)
**Age group (years)**			
60-69	263	118 (44.9)	44. 9 (38.7–51.0)
70-79	345	186 (53.9)	53.9 (48.5–59.3)
80 and above	259	182 (70.3)	70.3 (64.3–75.8)
**Gender**			
Male	330	171 (51.8)	51.8 (46.3–57.3)
Female	537	315 (58.7)	58.7 (54.4–62.9))
**Education level**			
Any education	751	400 (53.3)	53.3 (49.6–56.9)
No education	116	86 (74.1)	74.1 (65.2–81.8)
**Body Mass Index**			
Underweight/Normal (≤24.9)	397	210 (52.9)	52.9 (47.8–57.9)
Overweight (25.0 – 29.9)	289	159 (55.0)	55.0 (49.1–60.8)
Obese (30 and above)	153	97 (63.4)	63.4 (55.2–71.0)
**Hypertension**			
No	349	157 (45.0)	45.0 (39.7–50.4)
Yes	518	329 (63.5)	63.5 (59.2-67.7)
**Diabetes**			
No	604	345 (57.1)	57.1 (53.1–61.1)
Yes	263	141 (53.6)	53.6 (47.4–59.7)
**Mobility status**			
Mobility with support	613	275 (44.9)	44.9 (40.9–49.9)
Independently mobile	254	211 (83.1)	83.1 (77.9–87.5)
**Depression**			
Mild/None	672	313 (46.6)	46.5 (42.7–50.4)
Moderate	104	89 (85.6)	85.6 (77.3–91.7)
Severe	91	84 (92.3)	92.3 (84.8–96.8)

For peer review only - http://bmjopen.bmj.com/site/about/guidelines.xhtml

**History of fall in a year**			
No fall	625	318 (50.9)	50.9 (46.9–54.9)
Falls reported	242	168 (69.4)	69.4 (63.2–75.2)
**Visual impairment only**			
No	733	409 (55.8)	55.8 (52.1–59.4)
Yes	134	77 (57.5)	57.4 (48.6–66.0)
**Hearing impairment only**			
No	732	377 (51.5)	51.5 (47.8–55.2)
Yes	135	109 (80.1)	80.1 (73.1–87.0)
**Dual Sensory Impairment**			
No	817	440 (53.9)	53.9 (50.4–57.3)
Yes	50	46 (92.0)	92.0 (80.8–97.8)
**Multimorbidity**			
No	504	261	51.7 (47.3–56.2)
Yes	363	225	62.0 (56.8–67.0)
**Polypharmacy**			
No	706	382	54.1 (50.3–57.8)
Yes	161	104	64.6 (56.7–72.0)
**Total**	**867**	**486 (56.1)**	**56.1 (52.7–59.4)**

†Fear of falling was defined as a score of >= 9 on the Short Falls Efficacy Scale (SFES - International) questionnaire

Figure 1 shows the grades of FOF among the participants without any impairment, HI, VI, and DSI. The proportion of participants reporting a serious concern of falling was highest among those with DSI, followed by HI and VI (p<0.001).

Figure 1: Sensory loss and fear of falling among elderly in residential care.

Risk factors for FOF

Table 2 shows the association between FOF and various risk factors. FOF was significantly associated with older age, high BMI, and lack of education. The elderly residents with dual sensory impairment were eleven times more likely to have FOF (OR: 11.43; 95% CI: 3.15–41.41, Table 2) than those with no sensory impairment. HI was significantly associated with FOF (OR: 3.34; 95% CI: 1.96–5.72); however, VI (OR: 1.51; 95% CI: 0.95–2.40) was not significantly (p=0.08) associated. Those with moderate depression were seven times (OR: 6.86; 95% CI: 3.70–12.70) more likely to have a FOF, and those with severe depression were eight times more likely (OR:8.13; 95% CI: 3.50–18.88). A history of falls over the past year was also associated with an increase in FOF (OR: 1.53; 95% CI: 1.03–2.26). Those needing assistance for mobility were five times more likely to have FOF (OR: 4.84; 95% CI: 3.1–

For peer review only

7.44) compared to those with independent mobility. Polypharmacy and multimorbidity were not associated with FOF.

Table 2: Multiple logistic regression analysis, assessing the factors associated with fear of falling in the elderly in residential care.

	**Adjusted Odds Ratio (95% CI)**	**p-value**
**Age group (years)**		
60-69	Reference	
70-79	1.27 (0.86–1.88)	0.233
80 and above	1.84 (1.16–2.92)	0.010
**Gender**		
Male	Reference	
Female	1.35 (0.95–1.94)	0.097
**Education**		
Any education	Reference	
No education	2.31 (1.34–3.99)	0.003
**Body Mass Index (BMI)**		
Normal	Reference	
Overweight	1.28 (0.87–1.87)	0.215
Obese	2.03 (1.26–3.27)	0.003
**Multimorbidity**		
No	Reference	
Yes	1.22 (0.85–1.78)	0.273
**Polypharmacy**		
No	Reference	
Yes	1.36 (0.85–2.17)	0.193
**Mobility status**		
Independent mobility	Reference	
Mobility with support/aid	4.84 (3.15–7.44)	<0.001
**Depression**		
None	Reference	
Moderate	6.86 (3.71–12.70)	<0.001
Severe	8.13 (3.50–18.88)	<0.001
**History of fall in last year**		
No	Reference	
Yes	1.53 (1.03–2.26)	0.033
**Dual Sensory Impairment**		
No	Reference	
Yes	11.43 (3.15–41.42)	<0.001
**Vision impairment only**		
No	Reference	
Yes	1.51 (0.95–2.40)	0.084
**Hearing impairment only**		
No	Reference	
Yes	3.35 (1.96–5.72)	<0.001

For peer review only

DISCUSSION

Over half of the elderly population in residential care facilities had a fear of falling. In addition, nearly 90% of those with hearing and vision impairment (DSI) reported FOF. Earlier studies done in India reported a lower prevalence of FOF compared to the present study.[6-8] The possible reasons for a higher prevalence in this study could be explained by the differences in the living conditions of the elderly residents. Earlier studies reported on the elderly population living in a community setting, whereas those in the present study lived in institutional settings. Reports from other countries have shown a higher prevalence of FOF among residents of institutions.[28] No previous studies have reported FOF among elderly residents in institutional care in India. One study reported 42% prevalence of FOF among the elderly in a hospital setting in India.[29]

Studies from other parts of the world have reported a higher prevalence of FOF among the elderly living in institutional settings than those living in the communities.[28 30 31] However, the results from studies done in high-income countries cannot be extrapolated to India. Nursing homes and homes for the aged in high-income countries are well-established, with clear protocols in terms of infrastructure requirements, dedicated personnel specialised in elderly care, and laws governing the quality of care. Homes for the aged in India are relatively new, and there are no standard minimum requirements for establishing such institutions.[32] The facilities, personnel, and scope of service vary greatly based on the paying status of residents and the leadership of the facilities.[32] Homes are largely unmonitored and are run by private and non-government entities.[32] Due to these differences, the lifestyles of the elderly residents in homes in India and other high-income countries are not comparable.

The association between VI and FOF has been inconsistent across studies.[33-35] Some have reported a positive association,[36] while others have found no associations.[35 37 38] There was no association between VI and FOF in this study after adjusting for other covariates. A possible reason for the inconsistent associations across the studies is the differences in the definition used for VI. However, there could also be a small association, which most studies are underpowered to detect. We did find a positive association, but it was not statistically significant. It is also possible that the studies that reported an association between VI and FOF could have included those with DSI, which may have increased the association, as people with DSI were more likely to report FOF. Those with Dual Sensory Impairment (DSI)

For peer review only - http://bmjopen.bmj.com/site/about/guidelines.xhtml

For peer review only

had a substantially higher FOF compared to those with single sensory loss, either VI or HI. A recent study on the elderly population in nursing homes in India also reported a higher prevalence of FOF among those with dual sensory loss.[28]

The studies on eye health do not include the assessment of hearing as a routine procedure. We found that approximately 90% of those with DSI reported FOF, highlighting the need to assess both hearing status and eye health in the elderly population for adequate care and when carrying out research studies. Finally, though earlier studies reported a higher prevalence of FOF among women, there was no significant association with gender in this study.[39] FOF is associated independently with vision impairment and hearing impairment.[34] A recent study reported a higher prevalence of FOF among nursing home residents with sensory loss,[28] as over half of them had FOF.[28] A study by Murphy et al. reported that older people who had restricted their daily activities out of fear of falling had a higher burden of depressive symptoms than those who only reported fear of falling.[40] A meta-analysis reported that untreated depression is significantly associated with an increased risk of falling, which in turn may lead to FOF.[27]

FOF was associated with depression consistent with other studies.[13 30 36 41-46] Causality is impossible to demonstrate; however, several pathways for FOF leading to depression could be hypothesized. FOF is associated with reduced physical activity in the elderly.[47] This is evident in the current study by a higher odds for FOF among those needing assistance for mobility. The reduced activity results in a more sedentary lifestyle, increasing social isolation and leading to depression. However, it is also possible that those with higher levels of depression tend to become sedentary and limit their social interaction and mobility.
In our earlier paper, we reported that those with DSI were ten times more likely to have depression compared to those with single sensory loss.[19] In the current study, hearing impairment was independently associated with FOF after adjusting for other covariates. The possible explanation for this finding could be the changes in gait balance due to hearing impairment, leading to an increased FOF. A greater FOF was reported among those with lower gait velocity.[48]

Independent mobility is a measure of physical function, and a loss of it is associated with falls and FOF.[49-51] In this study, those requiring assistance for their mobility were five times more likely to have FOF compared to those who had independent mobility. Poor

For peer review only - http://bmjopen.bmj.com/site/about/guidelines.xhtml

physical function may result in a loss of confidence among the elderly, resulting in an increased FOF.[50] In a longitudinal study, FOF was associated with a lack of independent mobility and poor physical performance at the end of two years.[14] This indicates the need to address FOF in the elderly population to prevent a decline in physical functions.

The association between falls and FOF is reported in a few studies in India.[7 37] FOF was higher among those with a history of falls in India.[7 33] Adverse consequences of falls in the elderly are also well-known, which in itself may lead to FOF in older adults. Fear in itself is an innate response that has an evolutionary importance and is a protective instinct. Similarly, a fear of falling could act as an instinctual defence mechanism to prevent falls, but only to a certain threshold.[31 52] Beyond this threshold, FOF could impair physical functioning and negatively impact the quality of life of the elderly.[31 52] A recent systematic review has concluded that FOF adversely impacts the quality of life of elderly residents in community settings and institutional care.[53]

Several interventions have been reported to address FOF in the elderly living in communities and nursing homes. These interventions include cognitive and behavioural interventions, occupational therapy, and physical exercises, including balance training, yoga, and tai chi.[43 54-60] In addition to addressing FOF, these interventions may also have a resonating effect, alleviating depression, leading to better quality of life, and promoting well-being in the elderly population.[61-63] However, studies on the impact of such interventions that address FOF are limited in India. The elderly residents living in institutional care present a unique opportunity where interventions can be applied to more individuals in one setting, even with limited resources, than the elderly population diffused across communities. Studies also indicate an association between FOF and cognitive decline,[36 64] which underpins the need to address FOF among the elderly.

This study benefits from a large cohort of elderly participants and a comprehensive assessment (inclusion of dual sensory impairment and depression). Some limitations include our decision to exclude participants with severe mobility issues and those with cognitive impairment, as indicated by the lower HSME scores. This may have led to an underestimation of the prevalence of FOF in our study. However, it is unlikely to have led to an inaccurate assessment of associations. Environmental and infrastructure factors also influence FOF, which were not evaluated in our study. A direct extrapolation of our result to

For peer review only - http://bmjopen.bmj.com/site/about/guidelines.xhtml

those who live in their communities is limited due to the differences in the types of residences and the activities of people. Moreover, while visual acuity assessment using the standard procedures used in the clinic, hearing was assessed using a questionnaire, which is not an objective assessment. This limitation could have led to an underestimation of the prevalence of HI in our study.

In conclusion, FOF is common among the elderly population in residential care in India. It is especially common among those with depression and dual sensory impairment. With the increase in the elderly population and the mushrooming of homes for the aged in urban areas, these findings are relevant for policy and healthcare service planning for the elderly. This study findings can help plan and execute appropriate interventions to address FOF in the elderly population, with the goal of promoting healthy and active ageing among these residents.

Acknowledgments: The authors thank the individuals for their participation in the study. Mr. Shashank Yellapragada, Rajesh Challa, Madhuri Bakki is acknowledged for their assistance in data collection. Ratnakar Yellapragada and Ms. Muni Rajya Lakshmi are acknowledged for her support with data management. The authors also thank Mr. Abhinav Sekar for his language inputs on earlier versions of the manuscript.

Competing interests: "We have read and understood BMJ policy on declaration of interests and declare that we have no competing interests."

Funding Support: This work was supported by Wellcome Trust/DBT India Alliance Fellowship [IA/CPHE/14/1/501506] awarded to Dr. Srinivas Marmamula and Hyderabad Eye Research Foundation (HERF), India.

Contributorship Statement: SM conceived the idea, designed and conducted the study, analyzed the data and wrote the manuscript. TRK, SBM and NRB were involved in data collection. JEK and DSF reviewed the earlier version of the manuscripts and provide the intellectual inputs.

Data sharing statement: No additional data available.

For peer review only

Patient/participant involvement: The study participants were not involved in setting the research question or the outcome measures, but they were involved in the of the intervention. Participants and their care givers were directly involved in the study procedures and for provision of follow-up care beyond the study.

REFERENCES

1. UN. World Population Prospects 2017 United Nations, Department of Economic and Social Affairs, Population Division (2017, 2017.
2. Agrawal S. Effect of living arrangements on health status of elderly in India. Asian Population Studies 2012;**8**(1):87-101 doi: 10.1080/17441730.2012.646842[published Online First: Epub Date]|.
3. Mane AB. Ageing in India: some social challenges to elderly care. J Gerontol Geriatr Res 2016;**5**(2):e136
4. Legters K. Fear of falling. Phys Ther 2002;**82**(3):264-72
5. Malini FM, Lourenco RA, Lopes CS. Prevalence of fear of falling in older adults, and its associations with clinical, functional and psychosocial factors: the Frailty in Brazilian Older People-Rio de Janeiro study. Geriatr Gerontol Int 2016;**16**(3):336-44 doi: 10.1111/ggi.12477[published Online First: Epub Date]|.
6. Mane AB, Sanjana T, Patil PR, Sriniwas T. Prevalence and correlates of fear of falling among elderly population in urban area of Karnataka, India. Journal of mid-life health 2014;**5**(3):150-5 doi: 10.4103/0976-7800.141224[published Online First: Epub Date]|.
7. Pitchai P, Dedhia HB, Bhandari N, Krishnan D, D'Souza NRJ, Bellara JM. Prevalence, risk factors, circumstances for falls and level of functional independence among geriatric population - A descriptive study. Indian journal of public health 2019;**63**(1):21-26 doi: 10.4103/ijph.IJPH_332_17[published Online First: Epub Date]|.
8. Dhavale S, Singh S, Parasher RK. Factors Associated with Fear of Falls in Indian Elderly: A Systematic Review. Indian Journal of Public Health Research & Development 2019;**10**(10)
9. Dukyoo J, Juhee L, Lee SM. A meta-analysis of fear of falling treatment programs for the elderly. West J Nurs Res 2009;**31**(1):6-16 doi: 10.1177/0193945908320466[published Online First: Epub Date]|.
10. Shirooka H, Nishiguchi S, Fukutani N, et al. Cognitive impairment is associated with the absence of fear of falling in community-dwelling frail older adults. Geriatr Gerontol Int 2017;**17**(2):232-38 doi: 10.1111/ggi.12702[published Online First: Epub Date]|.
11. Auais M, French S, Alvarado B, Pirkle C, Belanger E, Guralnik J. Fear of Falling Predicts Incidence of Functional Disability 2 Years Later: A Perspective From an International Cohort Study. The journals of gerontology. Series A, Biological sciences and medical sciences 2018;**73**(9):1212-15 doi: 10.1093/gerona/glx237[published Online First: Epub Date]|.
12. Jung D. Fear of falling in older adults: comprehensive review. Asian Nurs Res (Korean Soc Nurs Sci) 2008;**2**(4):214-22 doi: 10.1016/S1976-1317(09)60003-7[published Online First: Epub Date]|.
13. Mishra N, Mishra AK, Bidija M. A study on correlation between depression, fear of fall and quality of life in elderly individuals. Int J Res Med Sci 2017;**5**(4):1456

For peer review only - http://bmjopen.bmj.com/site/about/guidelines.xhtml

14. Liu M, Hou T, Li Y, et al. Fear of falling is as important as multiple previous falls in terms of limiting daily activities: a longitudinal study. BMC Geriatr 2021;**21**(1):350 doi: 10.1186/s12877-021-02305-8[published Online First: Epub Date]|.
15. Scheffer AC, Schuurmans MJ, van Dijk N, van der Hooft T, de Rooij SE. Fear of falling: measurement strategy, prevalence, risk factors and consequences among older persons. Age and ageing 2008;**37**(1):19-24 doi: 10.1093/ageing/afm169[published Online First: Epub Date]|.
16. Marmamula S, Barrenkala NR, Challa R, et al. Hyderabad Ocular Morbidity in Elderly Study (HOMES) - Rationale, Study Design and Methodology. Ophthalmic Epidemiol 2020;**27**(1):83-92 doi: 10.1080/09286586.2019.1683867[published Online First: Epub Date]|.
17. Marmamula S, Barrenakala NR, Challa R, et al. Prevalence and risk factors for visual impairment among elderly residents in 'homes for the aged' in India: the Hyderabad Ocular Morbidity in Elderly Study (HOMES). Br J Ophthalmol 2021;**105**(1):32-36 doi: 10.1136/bjophthalmol-2019-315678[published Online First: Epub Date]|.
18. Marmamula S, Barrenkala NR, Challa R, et al. Falls and visual impairment among elderly residents in 'homes for the aged' in India. Sci Rep 2020;**10**(1):13389 doi: 10.1038/s41598-020-70066-2[published Online First: Epub Date]|.
19. Marmamula S, Kumbham TR, Modepalli SB, Barrenkala NR, Yellapragada R, Shidhaye R. Depression, combined visual and hearing impairment (dual sensory impairment): a hidden multi-morbidity among the elderly in Residential Care in India. Sci Rep 2021;**11**(1):16189 doi: 10.1038/s41598-021-95576-5[published Online First: Epub Date]|.
20. Marmamula S, Barrenkala NR, Rajesh C, et al. Hyderabad Ocular Morbidity in Elderly Study (HOMES) – Rationale, Study design and Methodology. Ophthalmic Epidemiol 2019((In press)) doi: 10.1080/09286586.2019.1683867[published Online First: Epub Date]|.
21. Folstein MF, Folstein SE, McHugh PR. "Mini-mental state". A practical method for grading the cognitive state of patients for the clinician. J Psychiatr Res 1975;**12**(3):189-98
22. Kroenke K, Spitzer RL, Williams JB, Lowe B. The Patient Health Questionnaire Somatic, Anxiety, and Depressive Symptom Scales: a systematic review. Gen Hosp Psychiatry 2010;**32**(4):345-59 doi: 10.1016/j.genhosppsych.2010.03.006[published Online First: Epub Date]|.
23. Ventry IM, Weinstein BE. The hearing handicap inventory for the elderly: a new tool. Ear Hear 1982;**3**(3):128-34
24. Kempen GI, Yardley L, van Haastregt JC, et al. The Short FES-I: a shortened version of the falls efficacy scale-international to assess fear of falling. Age and ageing 2008;**37**(1):45-50 doi: 10.1093/ageing/afm157[published Online First: Epub Date]|.
25. Tinetti ME, Richman D, Powell L. Falls efficacy as a measure of fear of falling. Journal of gerontology 1990;**45**(6):P239-P43
26. StataCorp. Stata Statistical Software: Release 14. College Station, TX: StataCorp LP., 2014.
27. Deandrea S, Lucenteforte E, Bravi F, Foschi R, La Vecchia C, Negri E. Risk factors for falls in community-dwelling older people: a systematic review and meta-analysis. Epidemiology 2010;**21**(5):658-68 doi: 10.1097/EDE.0b013e3181e89905[published Online First: Epub Date]|.
28. Lach HW, Lozano AJ, Hanlon AL, Cacchione PZ. Fear of falling in sensory impaired nursing home residents. Aging Ment Health 2020;**24**(3):474-80 doi: 10.1080/13607863.2018.1537359[published Online First: Epub Date]|.

For peer review only - http://bmjopen.bmj.com/site/about/guidelines.xhtml

29. Dhar M, Kaeley N, Mahala P, Saxena V, Pathania M. The Prevalence and Associated Risk Factors of Fear of Fall in the Elderly: A Hospital-Based, Cross-Sectional Study. Cureus 2022;**14**(3):e23479 doi: 10.7759/cureus.23479[published Online First: Epub Date]|.
30. Chou K-L, Yeung F, Wong E. Fear of falling and depressive symptoms in Chinese elderly living in nursing homes: fall efficacy and activity level as mediator or moderator? Aging & mental health 2005;**9**(3):255-61
31. Gillespie SM, Friedman SM. Fear of falling in new long-term care enrollees. Journal of the American Medical Directors Association 2007;**8**(5):307-13
32. Liebig PS. Old-age homes and services: old and new approaches to aged care. Journal of aging & social policy 2003;**15**(2-3):159-78 doi: 10.1300/J031v15n02_10[published Online First: Epub Date]|.
33. Subramanian MS, Singh V, Chatterjee P, Dwivedi SN, Dey AB. Prevalence and predictors of falls in a health-seeking older population: An outpatient-based study. Aging Med (Milton) 2020;**3**(1):25-31 doi: 10.1002/agm2.12096[published Online First: Epub Date]|.
34. White UE, Black AA, Wood JM, Delbaere K. Fear of falling in vision impairment. Optom Vis Sci 2015;**92**(6):730-5 doi: 10.1097/OPX.0000000000000596[published Online First: Epub Date]|.
35. Donoghue OA, Ryan H, Duggan E, et al. Relationship between fear of falling and mobility varies with visual function among older adults. Geriatr Gerontol Int 2014;**14**(4):827-36 doi: 10.1111/ggi.12174[published Online First: Epub Date]|.
36. Vo THM, Nakamura K, Seino K, Nguyen HTL, Van Vo T. Fear of falling and cognitive impairment in elderly with different social support levels: findings from a community survey in Central Vietnam. BMC Geriatr 2020;**20**(1):141 doi: 10.1186/s12877-020-01533-8[published Online First: Epub Date]|.
37. Friedman SM, Munoz B, West SK, Rubin GS, Fried LP. Falls and fear of falling: which comes first? A longitudinal prediction model suggests strategies for primary and secondary prevention. Journal of the American Geriatrics Society 2002;**50**(8):1329-35
38. Deshpande N, Metter EJ, Lauretani F, Bandinelli S, Guralnik J, Ferrucci L. Activity restriction induced by fear of falling and objective and subjective measures of physical function: a prospective cohort study. Journal of the American Geriatrics Society 2008;**56**(4):615-20 doi: 10.1111/j.1532-5415.2007.01639.x[published Online First: Epub Date]|.
39. Gazibara T, Kurtagic I, Kisic-Tepavcevic D, et al. Falls, risk factors and fear of falling among persons older than 65 years of age. Psychogeriatrics : the official journal of the Japanese Psychogeriatric Society 2017;**17**(4):215-23 doi: 10.1111/psyg.12217[published Online First: Epub Date]|.
40. Murphy SL, Williams CS, Gill TM. Characteristics associated with fear of falling and activity restriction in community-living older persons. Journal of the American Geriatrics Society 2002;**50**(3):516-20 doi: 10.1046/j.1532-5415.2002.50119.x[published Online First: Epub Date]|.
41. van Haastregt JC, Zijlstra GR, van Rossum E, van Eijk JTM, Kempen GI. Feelings of anxiety and symptoms of depression in community-living older persons who avoid activity for fear of falling. The American Journal of Geriatric Psychiatry 2008;**16**(3):186-93
42. Cumming RG, Salkeld G, Thomas M, Szonyi G. Prospective study of the impact of fear of falling on activities of daily living, SF-36 scores, and nursing home admission. The Journals of Gerontology Series A: Biological Sciences and Medical Sciences 2000;**55**(5):M299-M305

For peer review only

For peer review only - http://bmjopen.bmj.com/site/about/guidelines.xhtml

43. Huang TT, Chung ML, Chen FR, Chin YF, Wang BH. Evaluation of a combined cognitive-behavioural and exercise intervention to manage fear of falling among elderly residents in nursing homes. Aging Ment Health 2016;**20**(1):2-12 doi: 10.1080/13607863.2015.1020411[published Online First: Epub Date]|.
44. Berkman LF, Sekher T, Capistrant B, Zheng Y. Social networks, family, and care giving among older adults in India. Aging in Asia: Findings from new and emerging data initiatives: National academies Press (US), 2012.
45. Singh L, Singh PK, Arokiasamy P. Social Network and Mental Health Among Older Adults in Rural Uttar Pradesh, India: A Cross-Sectional Study. J Cross Cult Gerontol 2016;**31**(2):173-92 doi: 10.1007/s10823-016-9286-0[published Online First: Epub Date]|.
46. Hajek A, Bock JO, Konig HH. Psychological correlates of fear of falling: Findings from the German Aging Survey. Geriatr Gerontol Int 2018;**18**(3):396-406 doi: 10.1111/ggi.13190[published Online First: Epub Date]|.
47. Dsouza SA, Rajashekar B, Dsouza H, Kumar K. Falls in Indian older adults: a barrier to active ageing. Asian J Gerontol Geriatr 2014;**9**(1):1-8
48. Reelick MF, van Iersel MB, Kessels RP, Rikkert MG. The influence of fear of falling on gait and balance in older people. Age and ageing 2009;**38**(4):435-40 doi: 10.1093/ageing/afp066[published Online First: Epub Date]|.
49. Ayoubi F, Launay C, Annweiler C, Fantino B, Kabeshova A, Beauchet O. Fear of falling, falls, and gait variability in older community-dwelling individuals: is there an association? Journal of the American Geriatrics Society 2013;**61**(7):1236-8 doi: 10.1111/jgs.12350[published Online First: Epub Date]|.
50. Brouwer B, Musselman K, Culham E. Physical function and health status among seniors with and without a fear of falling. Gerontology 2004;**50**(3):135-41
51. Jefferis BJ, Iliffe S, Kendrick D, et al. How are falls and fear of falling associated with objectively measured physical activity in a cohort of community-dwelling older men? BMC Geriatr 2014;**14**:114 doi: 10.1186/1471-2318-14-114[published Online First: Epub Date]|.
52. Murphy SL, Dubin JA, Gill TM. The development of fear of falling among community-living older women: predisposing factors and subsequent fall events. The Journals of Gerontology Series A: Biological Sciences and Medical Sciences 2003;**58**(10):M943-M47
53. Schoene D, Heller C, Aung YN, Sieber CC, Kemmler W, Freiberger E. A systematic review on the influence of fear of falling on quality of life in older people: is there a role for falls? Clinical interventions in aging 2019;**14**:701-19 doi: 10.2147/CIA.S197857[published Online First: Epub Date]|.
54. Tennstedt S, Howland J, Lachman M, Peterson E, Kasten L, Jette A. A randomized, controlled trial of a group intervention to reduce fear of falling and associated activity restriction in older adults. The Journals of Gerontology Series B: Psychological Sciences and Social Sciences 1998;**53**(6):P384-P92
55. Walker JE, Howland J. Falls and fear of falling among elderly persons living in the community: occupational therapy interventions. Am J Occup Ther 1991;**45**(2):119-22 doi: 10.5014/ajot.45.2.119[published Online First: Epub Date]|.
56. Zhang J-G, Ishikawa-Takata K, Yamazaki H, Morita T, Ohta T. The effects of Tai Chi Chuan on physiological function and fear of falling in the less robust elderly: an intervention study for preventing falls. Archives of gerontology and geriatrics 2006;**42**(2):107-16

For peer review only - http://bmjopen.bmj.com/site/about/guidelines.xhtml

57. Sattin RW, Easley KA, Wolf SL, Chen Y, Kutner MH. Reduction in fear of falling through intense tai chi exercise training in older, transitionally frail adults. Journal of the American Geriatrics Society 2005;**53**(7):1168-78
58. Liu TW, Ng GYF, Ng SSM. Effectiveness of a combination of cognitive behavioral therapy and task-oriented balance training in reducing the fear of falling in patients with chronic stroke: study protocol for a randomized controlled trial. Trials 2018;**19**(1):168 doi: 10.1186/s13063-018-2549-z[published Online First: Epub Date]|.
59. Jung D, Lee J, Lee S-M. A meta-analysis of fear of falling treatment programs for the elderly. Western journal of nursing research 2009;**31**(1):6-16
60. Sjosten N, Vaapio S, Kivela SL. The effects of fall prevention trials on depressive symptoms and fear of falling among the aged: a systematic review. Aging Ment Health 2008;**12**(1):30-46 doi: 10.1080/13607860701366079[published Online First: Epub Date]|.
61. Chobe S, Chobe M, Metri K, Patra SK, Nagaratna R. Impact of Yoga on cognition and mental health among elderly: A systematic review. Complementary therapies in medicine 2020;**52**:102421 doi: 10.1016/j.ctim.2020.102421[published Online First: Epub Date]|.
62. Cramer H, Lauche R, Langhorst J, Dobos G. Yoga for depression: a systematic review and meta-analysis. Depress Anxiety 2013;**30**(11):1068-83 doi: 10.1002/da.22166[published Online First: Epub Date]|.
63. Zettergren KK, Lubeski JM, Viverito JM. Effects of a yoga program on postural control, mobility, and gait speed in community-living older adults: a pilot study. J Geriatr Phys Ther 2011;**34**(2):88-94 doi: 10.1519/JPT.0b013e31820aab53[published Online First: Epub Date]|.
64. Noh HM, Roh YK, Song HJ, Park YS. Severe Fear of Falling Is Associated With Cognitive Decline in Older Adults: A 3-Year Prospective Study. J Am Med Dir Assoc 2019;**20**(12):1540-47 doi: 10.1016/j.jamda.2019.06.008[published Online First: Epub Date]|.

For peer review only

For peer review only - http://bmjopen.bmj.com/site/about/guidelines.xhtml

For peer review only

For peer review only - http://bmjopen.bmj.com/site/about/guidelines.xhtml

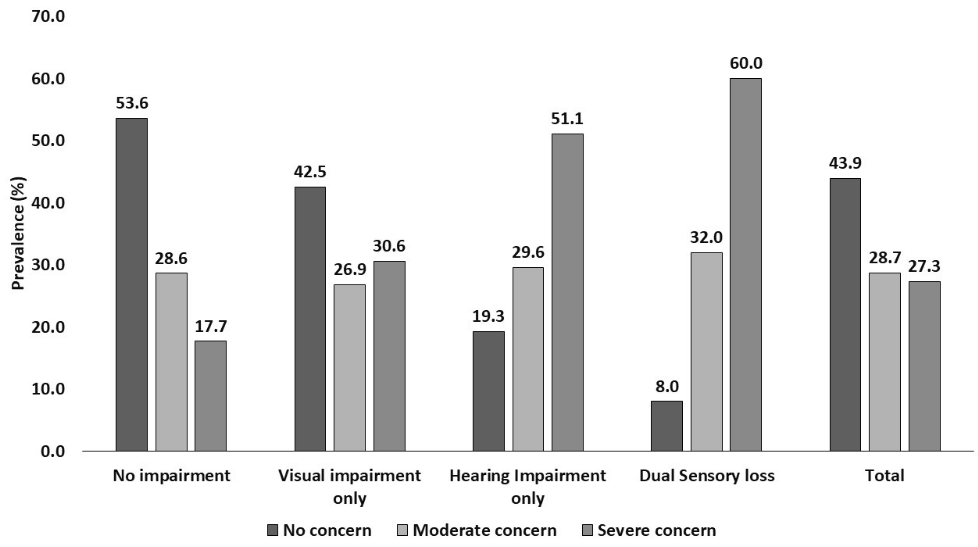

Sensory loss and fear of falling among elderly in residential care.

338x190mm (96 x 96 DPI)

STROBE 2007 (v4) Statement—Checklist of items that should be included in reports of *cross-sectional studies*

Section/Topic	Item #	Recommendation	Reported on page #
**Title and abstract**	1	(*a*) Indicate the study's design with a commonly used term in the title or the abstract	1
		(*b*) Provide in the abstract an informative and balanced summary of what was done and what was found	1
**Introduction**			
Background/rationale	2	Explain the scientific background and rationale for the investigation being reported	3
Objectives	3	State specific objectives, including any pre-specified hypotheses	3
**Methods**			
Study design	4	Present key elements of study design early in the paper	3
Setting	5	Describe the setting, locations, and relevant dates, including periods of recruitment, exposure, follow-up, and data collection	4,5
Participants	6	(*a*) Give the eligibility criteria, and the sources and methods of selection of participants	5,
Variables	7	Clearly define all outcomes, exposures, predictors, potential confounders, and effect modifiers. Give diagnostic criteria, if applicable	6,7
Data sources/ measurement	8*	For each variable of interest, give sources of data and details of methods of assessment (measurement). Describe comparability of assessment methods if there is more than one group	6,7
Bias	9	Describe any efforts to address potential sources of bias	NA
Study size	10	Explain how the study size was arrived at	5
Quantitative variables	11	Explain how quantitative variables were handled in the analyses. If applicable, describe which groupings were chosen and why	7
Statistical methods	12	(*a*) Describe all statistical methods, including those used to control for confounding	7
		(*b*) Describe any methods used to examine subgroups and interactions	7
		(*c*) Explain how missing data were addressed	NA
		(*d*) If applicable, describe analytical methods taking account of sampling strategy	NA
		(*e*) Describe any sensitivity analyses	NA
**Results**			

For peer review only

Participants	13*	(a) Report numbers of individuals at each stage of study—eg numbers potentially eligible, examined for eligibility, confirmed eligible, included in the study, completing follow-up, and analysed	NA
		(b) Give reasons for non-participation at each stage	NA
		(c) Consider use of a flow diagram	NA
Descriptive data	14*	(a) Give characteristics of study participants (eg demographic, clinical, social) and information on exposures and potential confounders	7
		(b) Indicate number of participants with missing data for each variable of interest	NA
Outcome data	15*	Report numbers of outcome events or summary measures	7
Main results	16	(*a*) Give unadjusted estimates and, if applicable, confounder-adjusted estimates and their precision (eg, 95% confidence interval). Make clear which confounders were adjusted for and why they were included	7
		(*b*) Report category boundaries when continuous variables were categorized	7
		(*c*) If relevant, consider translating estimates of relative risk into absolute risk for a meaningful time period	7
Other analyses	17	Report other analyses done—eg analyses of subgroups and interactions, and sensitivity analyses	7,8
**Discussion**			
Key results	18	Summarise key results with reference to study objectives	11,12
Limitations	19	Discuss limitations of the study, taking into account sources of potential bias or imprecision. Discuss both direction and magnitude of any potential bias	12,13
Interpretation	20	Give a cautious overall interpretation of results considering objectives, limitations, multiplicity of analyses, results from similar studies, and other relevant evidence	13
Generalisability	21	Discuss the generalisability (external validity) of the study results	13
**Other information**			
Funding	22	Give the source of funding and the role of the funders for the present study and, if applicable, for the original study on which the present article is based	14

*Give information separately for cases and controls in case-control studies and, if applicable, for exposed and unexposed groups in cohort and cross-sectional studies.

Note: An Explanation and Elaboration article discusses each checklist item and gives methodological background and published examples of transparent reporting. The STROBE checklist is best used in conjunction with this article (freely available on the Web sites of PLoS Medicine at http://www.plosmedicine.org/, Annals of Internal Medicine at http://www.annals.org/, and Epidemiology at http://www.epidem.com/). Information on the STROBE Initiative is available at www.strobe-statement.org.

For peer review only

BMJ Open

A cross sectional study of prevalence and correlates of fear of falling in the older people in residential care in India – the Hyderabad Ocular Morbidity in Elderly Study

Journal:	*BMJ Open*
Manuscript ID	bmjopen-2023-080973.R1
Article Type:	Original research
Date Submitted by the Author:	07-Mar-2024
Complete List of Authors:	Marmamula, Srinivas; LV Prasad Eye Institute, GPR ICARE; University of New South Wales<br>Kumbham, Thirupathi Reddy; LV Prasad Eye Institute, GPR ICARE<br>Modepalli, Satya Brahmanandam ; LV Prasad Eye Institute, Gullapalli Pratibha Rao International Centre for Advancement of Rural Eye care<br>Barrenkala, Navya Rekha; LV Prasad Eye Institute<br>Keeffe, Jill; LV Prasad Eye Institute, GPR ICARE<br>Friedman, David; Massachusetts Eye and Ear Infirmary
**Primary Subject Heading**:	Geriatric medicine
Secondary Subject Heading:	Epidemiology, Ophthalmology, Public health
Keywords:	Aging, EPIDEMIOLOGY, GERIATRIC MEDICINE, PUBLIC HEALTH

SCHOLARONE™
Manuscripts

I, the Submitting Author has the right to grant and does grant on behalf of all authors of the Work (as defined in the below author licence), an exclusive licence and/or a non-exclusive licence for contributions from authors who are: i) UK Crown employees; ii) where BMJ has agreed a CC-BY licence shall apply, and/or iii) in accordance with the terms applicable for US Federal Government officers or employees acting as part of their official duties; on a worldwide, perpetual, irrevocable, royalty-free basis to BMJ Publishing Group Ltd ("BMJ") its licensees and where the relevant Journal is co-owned by BMJ to the co-owners of the Journal, to publish the Work in this journal and any other BMJ products and to exploit all rights, as set out in our licence.

The Submitting Author accepts and understands that any supply made under these terms is made by BMJ to the Submitting Author unless you are acting as an employee on behalf of your employer or a postgraduate student of an affiliated institution which is paying any applicable article publishing charge ("APC") for Open Access articles. Where the Submitting Author wishes to make the Work available on an Open Access basis (and intends to pay the relevant APC), the terms of reuse of such Open Access shall be governed by a Creative Commons licence – details of these licences and which Creative Commons licence will apply to this Work are set out in our licence referred to above.

Other than as permitted in any relevant BMJ Author's Self Archiving Policies, I confirm this Work has not been accepted for publication elsewhere, is not being considered for publication elsewhere and does not duplicate material already published. I confirm all authors consent to publication of this Work and authorise the granting of this licence.

For peer review only

A cross sectional study of prevalence and correlates of fear of falling in the older people in residential care in India – the Hyderabad Ocular Morbidity in Elderly Study

Srinivas Marmamula,[1,2,3,4,5*] Thirupathi Reddy K,[1,2] Satya Brahmanandam Modepalli, [1,2] Navya Rekha Barrenkala,[1] Jill E Keeffe,[1] David S Friedman[6]

[1] Allen Foster Community Eye Health Research Centre, Gullapalli Pratibha Rao International Centre for Advancement of Rural Eye care, L V Prasad Eye Institute, Hyderabad, India

[2] Brien Holden Institute of Optometry and Vision Science, L V Prasad Eye Institute, Hyderabad, India

[3] Wellcome Trust / Department of Biotechnology India Alliance, L V Prasad Eye Institute, Hyderabad, India

[4] School of Optometry and Vision Science, University of New South Wales, Sydney, Australia

[5] Harvard T.H. Chan School of Public Health, Boston, MA, USA

[6] Massachusetts Eye and Ear, Harvard Medical School, Harvard Medical School, Department of Ophthalmology, Boston, USA

Running Title: Fear of falling in the older people in India

***Corresponding author:** Dr. Srinivas Marmamula, Gullapalli Pratibha Rao International Centre for Advancement of Rural Eye care, L V Prasad Eye Institute, Hyderabad, India. 500034. Email: sri.marmamula@lvpei.org

Total number of words: 3217 words excluding title page, abstract, acknowledgments, references, tables and figure legends

For peer review only - http://bmjopen.bmj.com/site/about/guidelines.xhtml

ABSTRACT

Objective: To report the prevalence and risk factors for the fear of falling (FOF) among older individuals living in residential care facilities in India.

Design: Cross sectional study

Setting: Homes for the aged centres in Hyderabad, India

Participants: The study included individuals aged ≥60 years from homes for the aged centres. The participants underwent a comprehensive eye examination in make-shift clinics in homes. Trained investigators collected the personal and demographic information of the participants using the Patient Health Questionnaire-9 and Hearing Handicap Inventory for Elderly questionnaire in the vernacular language. FOF was assessed using the Short Falls Efficacy Scale. The presence of hearing and vision impairment in the same individual was considered dual sensory impairment (DSI). A multiple logistic regression analysis was done to assess the factors associated with FOF.

Primary Outcome measure: Fear of Falling (FOF)

Results: In total, 867 participants were included from 41 homes for the aged centres in the analyses. The mean (±SD) age of the participants was 74.2 (±8.3) years (range: 60–96 years). The prevalence of FOF was 56.1% (95% CI:52.7–59.4; n=486. The multivariate analysis showed that those with DSI were eleven times more likely to report FOF than those with no impairment (OR:11.14; 95% CI:3.15–41.4.) Similarly, those with moderate depression were seven times more likely (OR:6.85; 95% CI:3.70–12.70), and those with severe depression were eight times (OR:8.13; 95% CI: 3.50–18.90) more likely to report FOF. A history of falls in the last year was also associated with increased odds for FOF (OR:1.52; 95% CI:1.03–2.26).

Conclusion: FOF is common among older individuals in residential care in India. Depression, falling in the previous year, and DSI were strongly associated with FOF. A cross disciplinary approach may be required to address FOF among the older people in residential care in India.

Keywords: Fear of falling, dual sensory impairment, depression, older people

For peer review only - http://bmjopen.bmj.com/site/about/guidelines.xhtml

ARTICLE SUMMARY:

Strengths and limitations of the study

- Hyderabad Ocular Morbidity in Elderly Study (HOMES) is a cross sectional study that included a large number of older people in residential care facilities in urban region in India.
- All participants underwent comprehensive assessments including visual acuity measurement, hearing assessment, depression, mobility and other assessments.
- Multiple logistic regression including vision impairment, hearing impairment and dual sensory impairment was done to assess independent predictors of fear of falling in the older population.
- Hearing assessment was based on a questionnaire, and this could have resulted in underestimating of prevalence of lower grades of hearing impairments.
- The results are generalizable only to the older population in residential care settings in urban India.

For peer review only

For peer review only - http://bmjopen.bmj.com/site/about/guidelines.xhtml

The proportion of the older people (people aged 60 years and older) in India's population is on the rise, and it is expected to reach 20% of the total population by 2050.[1] This demographic shift will dramatically change the nature of healthcare in India, and research on healthy ageing among the older population is needed. Such research can provide evidence for planning interventions to promote healthy and active ageing. Societal and lifestyle changes are sweeping through India, changing the living arrangements for the older population.[2 3] Multi-generational households are giving way to nuclear families and small households.[2 3] Under these changing circumstances, homes for the aged or residential care facilities are emerging and taking root rapidly.[2 3]

Fear of falling (FOF) is a term used to describe an individual's loss of confidence in their ability to maintain balance, resulting in a worry about falling.[4] The prevalence of FOF in the older population ranges from 29–92% with a history of falls and 12–65% without a history of falls.[5] In India, FOF is a common challenge affecting over a third of the older population[6-8] and affects their quality of life, physical well-being, and social functioning. Moreover, it is associated with depression,[9] cognitive decline, and incidence of functional disability in the older population and can predict future falls.[10 11] Several risk factors are associated with FOF, including vision impairment, hearing impairment, a history of falls, depression, and physical functioning.[12 13] A recent longitudinal study concluded that FOF is as detrimental as previous falls in limiting the daily activities among the older people.[14]

Studies on active ageing, falls, and FOF in older people are more common in high-income countries.[12 15] In India, such studies are still in a nascent stage, with very few studies reporting on FOF [6-8] and none reporting on the association of FOF with Dual Sensory Impairment (DSI, combined vision and hearing impairment) and depression among the older people residential care.

The Hyderabad Ocular Morbidity in Elderly Study (HOMES) is a large study conducted among older residents (aged ≥ 60 years) living in homes for the aged in Hyderabad, Telangana, India.[16] In addition to eye health, the study included an assessment of factors relevant to the health of older people, such as depression, falls, hearing impairment, and FOF.[16] Previous publications from this study reported on distance and near vision impairment, dual sensory loss, depression, prevalence, and risk factors for falls in the older

For peer review only - http://bmjopen.bmj.com/site/about/guidelines.xhtml

people.[17-19] In this paper, we report on the prevalence and associations of FOF with vision loss, hearing loss, dual sensory loss, and depression among the older people living in homes for the aged in India.

MATERIAL AND METHODS

Ethics approval

The protocol for the HOMES study was approved by the Institutional Review Board of the Hyderabad Eye Research Foundation, L V Prasad Eye Institute, Hyderabad, India (Reference number: LEC-08-16-073). The study was performed in accordance with the Declaration of Helsinki. Each participant provided written informed consent for their participation in the study. The data collection for this study was carried out during 2017-2019.

Sampling method and recruitment of the participants

The FOF was assessed as a part of a larger eye health and visual impairment study.[16] A sample size of 916 participants was required based on an anticipated prevalence of 15% for avoidable visual impairment, a 20% precision, a 25% non-response rate, and a design effect of 1.4 to account for clustering for a cluster size of 40 people. Based on anticipated prevalence of FOF of 33% as reported by a previous study, the sample required was 375 participants.[6] Of the 76 centres, 46 were selected (including five homes for the pilot) based on the proximity to a referral centre for eye care services and the willingness of the homes to participate in the study. Participants aged ≥60 years at the time of enumeration residing in the home for the aged for at least one month were included in the study.

Eye examination and other assessments

The examination procedures have been described in previous publications.[17-20] Briefly, monocular visual acuity (VA) was assessed at a three-meter distance under ambient illumination using a logMAR chart. The presenting and pinhole visual acuities were also assessed. Near vision was assessed using a logMAR chart at the standard distance of 40 cm. The anterior segment was examined using a portable slit lamp. A fundus examination and imaging were done for all participants and were graded by trained graders. Mobility was classified as independently mobile and mobile with assistance based on the participants' self-report and the interviewers' observations, including using a walking stick or assisted by other people. The participants who were bedridden or in a wheelchair were not included in the study, though they were examined and provided with the necessary services.

For peer review only - http://bmjopen.bmj.com/site/about/guidelines.xhtml

Non-clinical questionnaires

Trained investigators conducted detailed interviews with the participants using structured questionnaires.[16 20] All interviews were conducted before the eye examination. These included assessing the personal and demographic information (age, education level, marital status, and type of home) and systemic history (diabetes and hypertension). The Hindi Mini-Mental State Examination (HMSE) was used to assess cognitive status.[21] All the participants with an HMSE score of 20 or more were further interviewed and assessed for depression using the Patient Health Questionnaire-9 (PHQ-9).[22] The hearing was assessed using the Hearing Handicap Inventory for Elderly (HHIE-S) questionnaire with a hearing aid, if any.[23] Questionnaires were administered in the vernacular languages (Telugu or Hindi).

Fear of Falling (FOF) was assessed using the Short Falls Efficacy Scale (SFES - International) questionnaire.[24 25] This questionnaire has seven questions on commonly performed tasks, including self-care, physical activity, and participation in social events. The response grades included Not at All Concerned, Somewhat Concerned, Fairly Concerned, and Very Concerned. All the response grades were added to get a cumulative FOF score.[24] The history of previous falls was documented by asking, "Have you ever fallen on the floor in the last one year?" in the vernacular language (Telugu or Hindi). The response was recorded as 0=No, 1=Yes, and 3= Cannot remember.[18] Moreover, the participants were also asked if they had a fall in the last two weeks.

Definitions

a. Fear of Falling (FOF): A cumulative score ≥9 was considered as an individual with FOF. It was used as a dichotomous variable to define FOF. It was also graded as low (≤8), moderate (9-13), and high concern (14-28).
b. Vision Impairment (VI): Presenting distance visual acuity (VA) worse than 6/18 in the better eye, without hearing impairment (HI).
c. Hearing impairment (HI): It was defined as an HHIE Score <10 on the HHIE-S, without visual impairment (VI).
d. Dual Sensory Impairment (DSI): The presence of both VI and HI in an individual was considered DSI.

For peer review only - http://bmjopen.bmj.com/site/about/guidelines.xhtml

e. Depression: An individual with a cumulative score of ≥10 on the PHQ-9 was considered as having depression. It was graded as none/mild (0-9), moderate (10-14), and moderate-severe/severe (14-17).
f. Multimorbidity: It was defined as reporting two or more non-communicable diseases (NCDs).
g. Polypharmacy: It was defined as the use of five or more medications on a daily basis.

Patient and public involvement

The patients and the public were not involved in the design and conduct of the study.

Data management

Data analysis was conducted using Stata 14.0 (Stata Corp LP, College Station, TX).[26] For descriptive statistics, continuous variables were analysed using the Student's t-test, and the categorical variables were analysed using the chi-square test. The prevalence of FOF was estimated and presented with 95% confidence intervals (CI). In the multiple logistic regression analysis, FOF was used as a dichotomous outcome variable, and its association with personal and sociodemographic variables (age, gender, education), body mass index, multimorbidity, polypharmacy, depression, HI, VI, and DSI was evaluated. The variables were entered into the model one at a time, and the selection of covariates for the model was based on previous studies reporting on FOF.[27] The Hosmer-Lemeshow goodness of fit test was used to assess the model fit. Variance inflation factors (VIF) were used to test for collinearity between the covariates after fitting a multiple regression model. The adjusted odds ratio (OR) with 95% confidence intervals (CI) was presented. Statistical significance was set at $p<0.05$ (two-tailed), and the exact p-values were reported.

RESULTS

Characteristics of the study participants

The personal and demographic characteristics of the participants have been described in previous publications.[19] The HOMES study included 1182 participants; this included 271 participants who were bedridden and 98 participants with HMSE scores worse than 20.
Of the remaining 867 participants included in the analyses, 537 (61.6%) were women, 116 (13.4%) had no education, 518 (59.8%) reported hypertension, and 263 (30.3%) reported diabetes. The mean (±SD) age of the participants was 74.2 (±8.3) years (range: 60–96 years). In terms of mobility status, 613 (70.7%) were independent, and the remaining 254 (29.3%)

For peer review only - http://bmjopen.bmj.com/site/about/guidelines.xhtml

needed assistance from others or used walking aids for mobility. In total, 363 (41.9%) participants had multimorbidity and 161 (18.6%) reported polypharmacy.

Sensory impairments

In total, 548 (63.2%) participants had no sensory loss, 134 (15.5%) had VI, 135 (15.6%) had HI, and 50 (5.8%) had DSI. Among those with VI, the cause of VI was cataract (44.0%; n=59), uncorrected refractive errors (35.5%; n=48), posterior capsular opacification (9%; n=12), and others (11%; n=15).

Prevalence of FOF

The prevalence of FOF (moderate + high concern) was 56.1% (95% CI: 52.7–59.4; n=486). In total, 381 (43.9%) participants reported no concern about falling, 249 (28.7%) had a moderate concern, and 237 (27.3%) reported a high concern. The prevalence of FOF varied with the nature of sensory loss, with the highest prevalence among those with DSI (92.0%), followed by HI (80.1%) and VI (57.5%). The prevalence of FOF among those with a history of falls was 69.4% (95% CI: 63.2–75.2) compared to 50.9% (95% CI: 46.9–54.9) among those without a history of falling. Those with multimorbidity had a significantly higher prevalence of FOF (Table 1).

Table 1: Personal, demographic, and health-related characteristics and prevalence of fear of falling (n=867).

	Total in the sample	Fear of Falling † n (row %)	Fear of Falling - Prevalence (95% CI)
**Age group (years)**			
60-69	263	118 (44.9)	44. 9 (38.7–51.0)
70-79	345	186 (53.9)	53.9 (48.5–59.3)
80 and above	259	182 (70.3)	70.3 (64.3–75.8)
**Gender**			
Male	330	171 (51.8)	51.8 (46.3–57.3)
Female	537	315 (58.7)	58.7 (54.4–62.9))
**Education level**			
Any education	751	400 (53.3)	53.3 (49.6–56.9)
No education	116	86 (74.1)	74.1 (65.2–81.8)
**Body Mass Index**			

For peer review only - http://bmjopen.bmj.com/site/about/guidelines.xhtml

Underweight/Normal (≤24.9)	397	210 (52.9)	52.9 (47.8–57.9)
Overweight (25.0 – 29.9)	289	159 (55.0)	55.0 (49.1–60.8)
Obese (30 and above)	153	97 (63.4)	63.4 (55.2–71.0)
**Hypertension**			
No	349	157 (45.0)	45.0 (39.7–50.4)
Yes	518	329 (63.5)	63.5 (59.2-67.7)
**Diabetes**			
No	604	345 (57.1)	57.1 (53.1–61.1)
Yes	263	141 (53.6)	53.6 (47.4–59.7)
**Mobility status**			
Independently mobile	613	275 (44.9)	44.9 (40.9–49.9)
Mobility with support	254	211 (83.1)	83.1 (77.9–87.5)
**Depression**			
Mild/None	672	313 (46.6)	46.5 (42.7–50.4)
Moderate	104	89 (85.6)	85.6 (77.3–91.7)
Severe	91	84 (92.3)	92.3 (84.8–96.8)
**History of fall in a year**			
No fall	625	318 (50.9)	50.9 (46.9–54.9)
Falls reported	242	168 (69.4)	69.4 (63.2–75.2)
**Visual impairment only**			
No	733	409 (55.8)	55.8 (52.1–59.4)
Yes	134	77 (57.5)	57.4 (48.6–66.0)
**Hearing impairment only**			
No	732	377 (51.5)	51.5 (47.8–55.2)
Yes	135	109 (80.1)	80.1 (73.1–87.0)
**Dual Sensory Impairment**			
No	817	440 (53.9)	53.9 (50.4–57.3)
Yes	50	46 (92.0)	92.0 (80.8–97.8)
**Multimorbidity**			
No	504	261	51.7 (47.3–56.2)
Yes	363	225	62.0 (56.8–67.0)
**Polypharmacy**			
No	706	382	54.1 (50.3–57.8)
Yes	161	104	64.6 (56.7–72.0)
**Total**	**867**	**486 (56.1)**	**56.1 (52.7–59.4)**

†Fear of falling was defined as a score of >= 9 on the Short Falls Efficacy Scale (SFES - International) questionnaire; ‡BMI data not available on 28 participants.

Figure 1 shows the grades of FOF among the participants without any impairment, HI, VI, and DSI. The proportion of participants reporting a serious concern of falling was highest among those with DSI, followed by HI and VI (p<0.001).

Figure 1: Sensory loss and fear of falling among older people in residential care.

For peer review only - http://bmjopen.bmj.com/site/about/guidelines.xhtml

Risk factors for FOF

Table 2 shows the association between FOF and various risk factors. FOF was significantly associated with older age, high BMI, and lack of education. The older participants with dual sensory impairment had eleven times higher odds of FOF (OR: 11.43; 95% CI: 3.15–41.41, Table 2) than those with no sensory impairment. HI was significantly associated with FOF (OR: 3.34; 95% CI: 1.96–5.72); however, VI (OR: 1.51; 95% CI: 0.95–2.40) was not significantly (p=0.08) associated. Those with moderate depression had seven times (OR: 6.86; 95% CI: 3.70–12.70) higher odds of FOF, and those with severe depression had eight times higher odds (OR:8.13; 95% CI: 3.50–18.88). A history of falls over the past year was also associated with an increase in FOF (OR: 1.53; 95% CI: 1.03–2.26). Those needing assistance for mobility had five times higher odds of FOF (OR: 4.84; 95% CI: 3.1–7.44) compared to those with independent mobility. Polypharmacy and multimorbidity were not associated with FOF.

Table 2: Multiple logistic regression analysis, assessing the factors associated with fear of falling in the older people in residential care.(n=839)

	**Adjusted Odds Ratio (95% CI)**	**p-value**
**Age group (years)**		
60-69	Reference	
70-79	1.27 (0.86–1.88)	0.233
80 and above	1.84 (1.16–2.92)	0.010
**Gender**		
Male	Reference	
Female	1.35 (0.95–1.94)	0.097
**Education**		
Any education	Reference	
No education	2.31 (1.34–3.99)	0.003
**Body Mass Index (BMI)**		
Normal	Reference	
Overweight	1.28 (0.87–1.87)	0.215
Obese	2.03 (1.26–3.27)	0.003
**Multimorbidity**		
No	Reference	
Yes	1.22 (0.85–1.78)	0.273
**Polypharmacy**		
No	Reference	
Yes	1.36 (0.85–2.17)	0.193
**Mobility status**		
Independent mobility	Reference	
Mobility with support/aid	4.84 (3.15–7.44)	<0.001
**Depression**		

For peer review only - http://bmjopen.bmj.com/site/about/guidelines.xhtml

None	Reference	
Moderate	6.86 (3.71–12.70)	<0.001
Severe	8.13 (3.50–18.88)	<0.001
**History of fall in last year**		
No	Reference	
Yes	1.53 (1.03–2.26)	0.033
**Dual Sensory Impairment**		
No	Reference	
Yes	11.43 (3.15–41.42)	<0.001
**Vision impairment only**		
No	Reference	
Yes	1.51 (0.95–2.40)	0.084
**Hearing impairment only**		
No	Reference	
Yes	3.35 (1.96–5.72)	<0.001

For peer review only

DISCUSSION

Over half of the older people in residential care facilities had a fear of falling. In addition, nearly 90% of those with hearing and vision impairment (DSI) reported FOF. Earlier studies done in India reported a lower prevalence of FOF compared to the present study.[6-8] The possible reasons for a higher prevalence in this study could be explained by the differences in the living conditions of the older residents. Earlier studies reported on the older population living in a community setting, whereas those in the present study lived in institutional settings. Reports from other countries have shown a higher prevalence of FOF among residents of institutions.[28] No previous studies have reported FOF among older residents in institutional care in India. One study reported 42% prevalence of FOF among the older people in a hospital setting in India.[29]

Studies from other parts of the world have reported a higher prevalence of FOF among the older people living in institutional settings than those living in the communities.[28 30 31] However, the results from studies done in high-income countries cannot be extrapolated to India. Nursing homes and homes for the aged in high-income countries are well-established, with clear protocols in terms of infrastructure requirements, dedicated personnel specialised in care for the older people and laws governing the quality of care. Homes for the aged in India are relatively new, and there are no standard minimum requirements for establishing such institutions.[32] The facilities, personnel, and scope of service vary greatly based on the paying status of residents and the leadership of the facilities.[32] Homes in India are largely

For peer review only - http://bmjopen.bmj.com/site/about/guidelines.xhtml

unmonitored and are run by private and non-government entities.[32] Due to these differences, the lifestyles of the older residents in homes in India and other high-income countries are not comparable.

The association between VI and FOF has been inconsistent across studies.[33-35] Some have reported a positive association,[36] while others have found no associations.[35 37 38] There was no association between VI and FOF in this study after adjusting for other covariates. A possible reason for the inconsistent associations across the studies is the differences in the definition used for VI. However, there could also be a small association, which most studies are underpowered to detect. We did find a positive association, but it was not statistically significant. It is also possible that the studies that reported an association between VI and FOF could have included those with DSI, which may have increased the association, as people with DSI were more likely to report FOF. Those with Dual Sensory Impairment (DSI) had a substantially higher FOF compared to those with single sensory loss, either VI or HI. A recent study on the older population in nursing homes in India also reported a higher prevalence of FOF among those with dual sensory loss.[28]

The studies on eye health do not include the assessment of hearing as a routine procedure. We found that approximately 90% of those with DSI reported FOF, highlighting the need to assess both hearing status and eye health in the older population for adequate care and when carrying out research studies. Finally, though earlier studies reported a higher prevalence of FOF among women, there was no significant association with gender in this study.[39] FOF is associated independently with vision impairment and hearing impairment.[34] A recent study reported a higher prevalence of FOF among nursing home residents with sensory loss,[28] as over half of them had FOF.[28] A study by Murphy et al. reported that older people who had restricted their daily activities out of fear of falling had a higher burden of depressive symptoms than those who only reported fear of falling.[40] A meta-analysis reported that untreated depression is significantly associated with an increased risk of falling, which in turn may lead to FOF.[27]

FOF was associated with depression consistent with other studies.[13 30 36 41-46] Causality is impossible to demonstrate; however, several pathways for FOF leading to depression could be hypothesized. FOF is associated with reduced physical activity in the older people.[47] This is evident in the current study by a higher odds for FOF among those needing assistance

For peer review only - http://bmjopen.bmj.com/site/about/guidelines.xhtml

for mobility. The reduced activity results in a more sedentary lifestyle, increasing social isolation and leading to depression. However, it is also possible that those with higher levels of depression tend to become sedentary and limit their social interaction and mobility.

In our earlier paper, we reported that those with DSI were ten times more likely to have depression compared to those with single sensory loss.[19] In the current study, hearing impairment was independently associated with FOF after adjusting for other covariates. The possible explanation for this finding could be the changes in gait balance due to hearing impairment, leading to an increased FOF. A greater FOF was reported among those with lower gait velocity.[48]

Independent mobility is a measure of physical function, and a loss of it is associated with falls and FOF.[49-51] In this study, those requiring assistance for their mobility were five times more likely to have FOF compared to those who had independent mobility. Poor physical function may result in a loss of confidence among the older people, resulting in an increased FOF.[50] In a longitudinal study, FOF was associated with a lack of independent mobility and poor physical performance at the end of two years.[14] This indicates the need to address FOF in the older population to prevent a decline in physical functions.

The association between falls and FOF is reported in a few studies in India.[7 37] FOF was higher among those with a history of falls in India.[7 33] Adverse consequences of falls in the older people are also well-known, which in itself may lead to FOF in older adults. Fear in itself is an innate response that has an evolutionary importance and is a protective instinct. Similarly, a fear of falling could act as an instinctual defence mechanism to prevent falls, but only to a certain threshold.[31 52] Beyond this threshold, FOF could impair physical functioning and negatively impact the quality of life of the older people.[31 52] A recent systematic review has concluded that FOF adversely impacts the quality of life of older residents in community settings and institutional care.[53]

Several interventions have been reported to address FOF in the older people living in communities and nursing homes. These interventions include cognitive and behavioural interventions, occupational therapy, and physical exercises, including balance training, yoga, and tai chi.[43 54-60] In addition to addressing FOF, these interventions may also have a resonating effect, alleviating depression, leading to better quality of life, and promoting well-

For peer review only - http://bmjopen.bmj.com/site/about/guidelines.xhtml

being in the older population.[61-63] However, studies on the impact of such interventions that address FOF are limited in India. The older residents living in institutional care present a unique opportunity where interventions can be applied to more individuals in one setting, even with limited resources, than the older population diffused across communities. Studies also indicate an association between FOF and cognitive decline,[36 64] which underpins the need to address FOF among older people.

This study benefits from a large cohort of older participants and a comprehensive assessment (inclusion of dual sensory impairment and depression). Some limitations include our decision to exclude participants with severe mobility issues and those with cognitive impairment, as indicated by the lower HSME scores. This may have led to an underestimation of the prevalence of FOF in our study. However, it is unlikely to have led to an inaccurate assessment of associations. Environmental and infrastructure factors also influence FOF, which were not evaluated in our study. A direct extrapolation of our result to those who live in their communities is limited due to the differences in the types of residences and the activities of people. Moreover, while visual acuity assessment using the standard procedures used in the clinic, hearing was assessed using a questionnaire, which is not an objective assessment. This limitation could have led to an underestimation of the prevalence of HI in our study. HOMES was an observational study hence causality cannot be established.

In conclusion, FOF is common among the older population in residential care in India. It is especially common among those with depression and dual sensory impairment. With the increase in the older population and the rapid increase in the number of homes for the aged in urban areas, these findings are relevant for policy and healthcare service planning for older people. This study findings can inform planners to develop appropriate interventions to address FOF in the older population, to promote healthy and active ageing among these residents.

Acknowledgments: The authors thank the individuals for their participation in the study. Mr. Shashank Yellapragada, Rajesh Challa, Madhuri Bakki is acknowledged for their assistance in data collection. Ratnakar Yellapragada and Ms. Muni Rajya Lakshmi are acknowledged for her support with data management. The authors also thank Mr. Abhinav Sekar for his language inputs on earlier versions of the manuscript.

For peer review only - http://bmjopen.bmj.com/site/about/guidelines.xhtml

Competing interests: "We have read and understood BMJ policy on declaration of interests and declare that we have no competing interests."

Funding Support: This work was supported by Wellcome Trust/DBT India Alliance Fellowship [IA/CPHE/14/1/501506] awarded to Dr. Srinivas Marmamula and Hyderabad Eye Research Foundation (HERF), India.

Contributorship Statement: SM conceived the idea, designed and conducted the study, analyzed the data and wrote the manuscript. TRK, SBM and NRB were involved in data collection and data management. JEK and DSF reviewed the earlier version of the manuscripts and provide the intellectual inputs.

Data sharing statement: No additional data are available.

For peer review only

REFERENCES

1. UN. World Population Prospects 2017 United Nations, Department of Economic and Social Affairs, Population Division (2017, 2017.
2. Agrawal S. Effect of living arrangements on health status of elderly in India. Asian Population Studies 2012;**8**(1):87-101 doi: 10.1080/17441730.2012.646842[published Online First: Epub Date]|.
3. Mane AB. Ageing in India: some social challenges to elderly care. J Gerontol Geriatr Res 2016;**5**(2):e136
4. Legters K. Fear of falling. Phys Ther 2002;**82**(3):264-72
5. Malini FM, Lourenco RA, Lopes CS. Prevalence of fear of falling in older adults, and its associations with clinical, functional and psychosocial factors: the Frailty in Brazilian Older People-Rio de Janeiro study. Geriatr Gerontol Int 2016;**16**(3):336-44 doi: 10.1111/ggi.12477[published Online First: Epub Date]|.
6. Mane AB, Sanjana T, Patil PR, Sriniwas T. Prevalence and correlates of fear of falling among elderly population in urban area of Karnataka, India. Journal of mid-life health 2014;**5**(3):150-5 doi: 10.4103/0976-7800.141224[published Online First: Epub Date]|.
7. Pitchai P, Dedhia HB, Bhandari N, Krishnan D, D'Souza NRJ, Bellara JM. Prevalence, risk factors, circumstances for falls and level of functional independence among geriatric population - A descriptive study. Indian journal of public health 2019;**63**(1):21-26 doi: 10.4103/ijph.IJPH_332_17[published Online First: Epub Date]|.
8. Dhavale S, Singh S, Parasher RK. Factors Associated with Fear of Falls in Indian Elderly: A Systematic Review. Indian Journal of Public Health Research & Development 2019;**10**(10)
9. Dukyoo J, Juhee L, Lee SM. A meta-analysis of fear of falling treatment programs for the elderly. West J Nurs Res 2009;**31**(1):6-16 doi: 10.1177/0193945908320466[published Online First: Epub Date]|.

For peer review only - http://bmjopen.bmj.com/site/about/guidelines.xhtml

10. Shirooka H, Nishiguchi S, Fukutani N, et al. Cognitive impairment is associated with the absence of fear of falling in community-dwelling frail older adults. Geriatr Gerontol Int 2017;**17**(2):232-38 doi: 10.1111/ggi.12702[published Online First: Epub Date]|.
11. Auais M, French S, Alvarado B, Pirkle C, Belanger E, Guralnik J. Fear of Falling Predicts Incidence of Functional Disability 2 Years Later: A Perspective From an International Cohort Study. The journals of gerontology. Series A, Biological sciences and medical sciences 2018;**73**(9):1212-15 doi: 10.1093/gerona/glx237[published Online First: Epub Date]|.
12. Jung D. Fear of falling in older adults: comprehensive review. Asian Nurs Res (Korean Soc Nurs Sci) 2008;**2**(4):214-22 doi: 10.1016/S1976-1317(09)60003-7[published Online First: Epub Date]|.
13. Mishra N, Mishra AK, Bidija M. A study on correlation between depression, fear of fall and quality of life in elderly individuals. Int J Res Med Sci 2017;**5**(4):1456
14. Liu M, Hou T, Li Y, et al. Fear of falling is as important as multiple previous falls in terms of limiting daily activities: a longitudinal study. BMC Geriatr 2021;**21**(1):350 doi: 10.1186/s12877-021-02305-8[published Online First: Epub Date]|.
15. Scheffer AC, Schuurmans MJ, van Dijk N, van der Hooft T, de Rooij SE. Fear of falling: measurement strategy, prevalence, risk factors and consequences among older persons. Age and ageing 2008;**37**(1):19-24 doi: 10.1093/ageing/afm169[published Online First: Epub Date]|.
16. Marmamula S, Barrenkala NR, Challa R, et al. Hyderabad Ocular Morbidity in Elderly Study (HOMES) - Rationale, Study Design and Methodology. Ophthalmic Epidemiol 2020;**27**(1):83-92 doi: 10.1080/09286586.2019.1683867[published Online First: Epub Date]|.
17. Marmamula S, Barrenakala NR, Challa R, et al. Prevalence and risk factors for visual impairment among elderly residents in 'homes for the aged' in India: the Hyderabad Ocular Morbidity in Elderly Study (HOMES). Br J Ophthalmol 2021;**105**(1):32-36 doi: 10.1136/bjophthalmol-2019-315678[published Online First: Epub Date]|.
18. Marmamula S, Barrenkala NR, Challa R, et al. Falls and visual impairment among elderly residents in 'homes for the aged' in India. Sci Rep 2020;**10**(1):13389 doi: 10.1038/s41598-020-70066-2[published Online First: Epub Date]|.
19. Marmamula S, Kumbham TR, Modepalli SB, Barrenkala NR, Yellapragada R, Shidhaye R. Depression, combined visual and hearing impairment (dual sensory impairment): a hidden multi-morbidity among the elderly in Residential Care in India. Sci Rep 2021;**11**(1):16189 doi: 10.1038/s41598-021-95576-5[published Online First: Epub Date]|.
20. Marmamula S, Barrenkala NR, Rajesh C, et al. Hyderabad Ocular Morbidity in Elderly Study (HOMES) – Rationale, Study design and Methodology. Ophthalmic Epidemiol 2019((In press)) doi: 10.1080/09286586.2019.1683867[published Online First: Epub Date]|.
21. Folstein MF, Folstein SE, McHugh PR. "Mini-mental state". A practical method for grading the cognitive state of patients for the clinician. J Psychiatr Res 1975;**12**(3):189-98
22. Kroenke K, Spitzer RL, Williams JB, Lowe B. The Patient Health Questionnaire Somatic, Anxiety, and Depressive Symptom Scales: a systematic review. Gen Hosp Psychiatry 2010;**32**(4):345-59 doi: 10.1016/j.genhosppsych.2010.03.006[published Online First: Epub Date]|.
23. Ventry IM, Weinstein BE. The hearing handicap inventory for the elderly: a new tool. Ear Hear 1982;**3**(3):128-34

For peer review only - http://bmjopen.bmj.com/site/about/guidelines.xhtml

24. Kempen GI, Yardley L, van Haastregt JC, et al. The Short FES-I: a shortened version of the falls efficacy scale-international to assess fear of falling. Age and ageing 2008;**37**(1):45-50 doi: 10.1093/ageing/afm157[published Online First: Epub Date]|.
25. Tinetti ME, Richman D, Powell L. Falls efficacy as a measure of fear of falling. Journal of gerontology 1990;**45**(6):P239-P43
26. StataCorp. Stata Statistical Software: Release 14. College Station, TX: StataCorp LP., 2014.
27. Deandrea S, Lucenteforte E, Bravi F, Foschi R, La Vecchia C, Negri E. Risk factors for falls in community-dwelling older people: a systematic review and meta-analysis. Epidemiology 2010;**21**(5):658-68 doi: 10.1097/EDE.0b013e3181e89905[published Online First: Epub Date]|.
28. Lach HW, Lozano AJ, Hanlon AL, Cacchione PZ. Fear of falling in sensory impaired nursing home residents. Aging Ment Health 2020;**24**(3):474-80 doi: 10.1080/13607863.2018.1537359[published Online First: Epub Date]|.
29. Dhar M, Kaeley N, Mahala P, Saxena V, Pathania M. The Prevalence and Associated Risk Factors of Fear of Fall in the Elderly: A Hospital-Based, Cross-Sectional Study. Cureus 2022;**14**(3):e23479 doi: 10.7759/cureus.23479[published Online First: Epub Date]|.
30. Chou K-L, Yeung F, Wong E. Fear of falling and depressive symptoms in Chinese elderly living in nursing homes: fall efficacy and activity level as mediator or moderator? Aging & mental health 2005;**9**(3):255-61
31. Gillespie SM, Friedman SM. Fear of falling in new long-term care enrollees. Journal of the American Medical Directors Association 2007;**8**(5):307-13
32. Liebig PS. Old-age homes and services: old and new approaches to aged care. Journal of aging & social policy 2003;**15**(2-3):159-78 doi: 10.1300/J031v15n02_10[published Online First: Epub Date]|.
33. Subramanian MS, Singh V, Chatterjee P, Dwivedi SN, Dey AB. Prevalence and predictors of falls in a health-seeking older population: An outpatient-based study. Aging Med (Milton) 2020;**3**(1):25-31 doi: 10.1002/agm2.12096[published Online First: Epub Date]|.
34. White UE, Black AA, Wood JM, Delbaere K. Fear of falling in vision impairment. Optom Vis Sci 2015;**92**(6):730-5 doi: 10.1097/OPX.0000000000000596[published Online First: Epub Date]|.
35. Donoghue OA, Ryan H, Duggan E, et al. Relationship between fear of falling and mobility varies with visual function among older adults. Geriatr Gerontol Int 2014;**14**(4):827-36 doi: 10.1111/ggi.12174[published Online First: Epub Date]|.
36. Vo THM, Nakamura K, Seino K, Nguyen HTL, Van Vo T. Fear of falling and cognitive impairment in elderly with different social support levels: findings from a community survey in Central Vietnam. BMC Geriatr 2020;**20**(1):141 doi: 10.1186/s12877-020-01533-8[published Online First: Epub Date]|.
37. Friedman SM, Munoz B, West SK, Rubin GS, Fried LP. Falls and fear of falling: which comes first? A longitudinal prediction model suggests strategies for primary and secondary prevention. Journal of the American Geriatrics Society 2002;**50**(8):1329-35
38. Deshpande N, Metter EJ, Lauretani F, Bandinelli S, Guralnik J, Ferrucci L. Activity restriction induced by fear of falling and objective and subjective measures of physical function: a prospective cohort study. Journal of the American Geriatrics Society 2008;**56**(4):615-20 doi: 10.1111/j.1532-5415.2007.01639.x[published Online First: Epub Date]|.
39. Gazibara T, Kurtagic I, Kisic-Tepavcevic D, et al. Falls, risk factors and fear of falling among persons older than 65 years of age. Psychogeriatrics : the official journal of the

For peer review only - http://bmjopen.bmj.com/site/about/guidelines.xhtml

Japanese Psychogeriatric Society 2017;**17**(4):215-23 doi: 10.1111/psyg.12217[published Online First: Epub Date]|.
40. Murphy SL, Williams CS, Gill TM. Characteristics associated with fear of falling and activity restriction in community-living older persons. Journal of the American Geriatrics Society 2002;**50**(3):516-20 doi: 10.1046/j.1532-5415.2002.50119.x[published Online First: Epub Date]|.
41. van Haastregt JC, Zijlstra GR, van Rossum E, van Eijk JTM, Kempen GI. Feelings of anxiety and symptoms of depression in community-living older persons who avoid activity for fear of falling. The American Journal of Geriatric Psychiatry 2008;**16**(3):186-93
42. Cumming RG, Salkeld G, Thomas M, Szonyi G. Prospective study of the impact of fear of falling on activities of daily living, SF-36 scores, and nursing home admission. The Journals of Gerontology Series A: Biological Sciences and Medical Sciences 2000;**55**(5):M299-M305
43. Huang TT, Chung ML, Chen FR, Chin YF, Wang BH. Evaluation of a combined cognitive-behavioural and exercise intervention to manage fear of falling among elderly residents in nursing homes. Aging Ment Health 2016;**20**(1):2-12 doi: 10.1080/13607863.2015.1020411[published Online First: Epub Date]|.
44. Berkman LF, Sekher T, Capistrant B, Zheng Y. Social networks, family, and care giving among older adults in India. Aging in Asia: Findings from new and emerging data initiatives: National academies Press (US), 2012.
45. Singh L, Singh PK, Arokiasamy P. Social Network and Mental Health Among Older Adults in Rural Uttar Pradesh, India: A Cross-Sectional Study. J Cross Cult Gerontol 2016;**31**(2):173-92 doi: 10.1007/s10823-016-9286-0[published Online First: Epub Date]|.
46. Hajek A, Bock JO, Konig HH. Psychological correlates of fear of falling: Findings from the German Aging Survey. Geriatr Gerontol Int 2018;**18**(3):396-406 doi: 10.1111/ggi.13190[published Online First: Epub Date]|.
47. Dsouza SA, Rajashekar B, Dsouza H, Kumar K. Falls in Indian older adults: a barrier to active ageing. Asian J Gerontol Geriatr 2014;**9**(1):1-8
48. Reelick MF, van Iersel MB, Kessels RP, Rikkert MG. The influence of fear of falling on gait and balance in older people. Age and ageing 2009;**38**(4):435-40 doi: 10.1093/ageing/afp066[published Online First: Epub Date]|.
49. Ayoubi F, Launay C, Annweiler C, Fantino B, Kabeshova A, Beauchet O. Fear of falling, falls, and gait variability in older community-dwelling individuals: is there an association? Journal of the American Geriatrics Society 2013;**61**(7):1236-8 doi: 10.1111/jgs.12350[published Online First: Epub Date]|.
50. Brouwer B, Musselman K, Culham E. Physical function and health status among seniors with and without a fear of falling. Gerontology 2004;**50**(3):135-41
51. Jefferis BJ, Iliffe S, Kendrick D, et al. How are falls and fear of falling associated with objectively measured physical activity in a cohort of community-dwelling older men? BMC Geriatr 2014;**14**:114 doi: 10.1186/1471-2318-14-114[published Online First: Epub Date]|.
52. Murphy SL, Dubin JA, Gill TM. The development of fear of falling among community-living older women: predisposing factors and subsequent fall events. The Journals of Gerontology Series A: Biological Sciences and Medical Sciences 2003;**58**(10):M943-M47
53. Schoene D, Heller C, Aung YN, Sieber CC, Kemmler W, Freiberger E. A systematic review on the influence of fear of falling on quality of life in older people: is there a

For peer review only - http://bmjopen.bmj.com/site/about/guidelines.xhtml

role for falls? Clinical interventions in aging 2019;**14**:701-19 doi: 10.2147/CIA.S197857[published Online First: Epub Date]|.
54. Tennstedt S, Howland J, Lachman M, Peterson E, Kasten L, Jette A. A randomized, controlled trial of a group intervention to reduce fear of falling and associated activity restriction in older adults. The Journals of Gerontology Series B: Psychological Sciences and Social Sciences 1998;**53**(6):P384-P92
55. Walker JE, Howland J. Falls and fear of falling among elderly persons living in the community: occupational therapy interventions. Am J Occup Ther 1991;**45**(2):119-22 doi: 10.5014/ajot.45.2.119[published Online First: Epub Date]|.
56. Zhang J-G, Ishikawa-Takata K, Yamazaki H, Morita T, Ohta T. The effects of Tai Chi Chuan on physiological function and fear of falling in the less robust elderly: an intervention study for preventing falls. Archives of gerontology and geriatrics 2006;**42**(2):107-16
57. Sattin RW, Easley KA, Wolf SL, Chen Y, Kutner MH. Reduction in fear of falling through intense tai chi exercise training in older, transitionally frail adults. Journal of the American Geriatrics Society 2005;**53**(7):1168-78
58. Liu TW, Ng GYF, Ng SSM. Effectiveness of a combination of cognitive behavioral therapy and task-oriented balance training in reducing the fear of falling in patients with chronic stroke: study protocol for a randomized controlled trial. Trials 2018;**19**(1):168 doi: 10.1186/s13063-018-2549-z[published Online First: Epub Date]|.
59. Jung D, Lee J, Lee S-M. A meta-analysis of fear of falling treatment programs for the elderly. Western journal of nursing research 2009;**31**(1):6-16
60. Sjosten N, Vaapio S, Kivela SL. The effects of fall prevention trials on depressive symptoms and fear of falling among the aged: a systematic review. Aging Ment Health 2008;**12**(1):30-46 doi: 10.1080/13607860701366079[published Online First: Epub Date]|.
61. Chobe S, Chobe M, Metri K, Patra SK, Nagaratna R. Impact of Yoga on cognition and mental health among elderly: A systematic review. Complementary therapies in medicine 2020;**52**:102421 doi: 10.1016/j.ctim.2020.102421[published Online First: Epub Date]|.
62. Cramer H, Lauche R, Langhorst J, Dobos G. Yoga for depression: a systematic review and meta-analysis. Depress Anxiety 2013;**30**(11):1068-83 doi: 10.1002/da.22166[published Online First: Epub Date]|.
63. Zettergren KK, Lubeski JM, Viverito JM. Effects of a yoga program on postural control, mobility, and gait speed in community-living older adults: a pilot study. J Geriatr Phys Ther 2011;**34**(2):88-94 doi: 10.1519/JPT.0b013e31820aab53[published Online First: Epub Date]|.
64. Noh HM, Roh YK, Song HJ, Park YS. Severe Fear of Falling Is Associated With Cognitive Decline in Older Adults: A 3-Year Prospective Study. J Am Med Dir Assoc 2019;**20**(12):1540-47 doi: 10.1016/j.jamda.2019.06.008[published Online First: Epub Date]|.

For peer review only - http://bmjopen.bmj.com/site/about/guidelines.xhtml

For peer review only

For peer review only - http://bmjopen.bmj.com/site/about/guidelines.xhtml

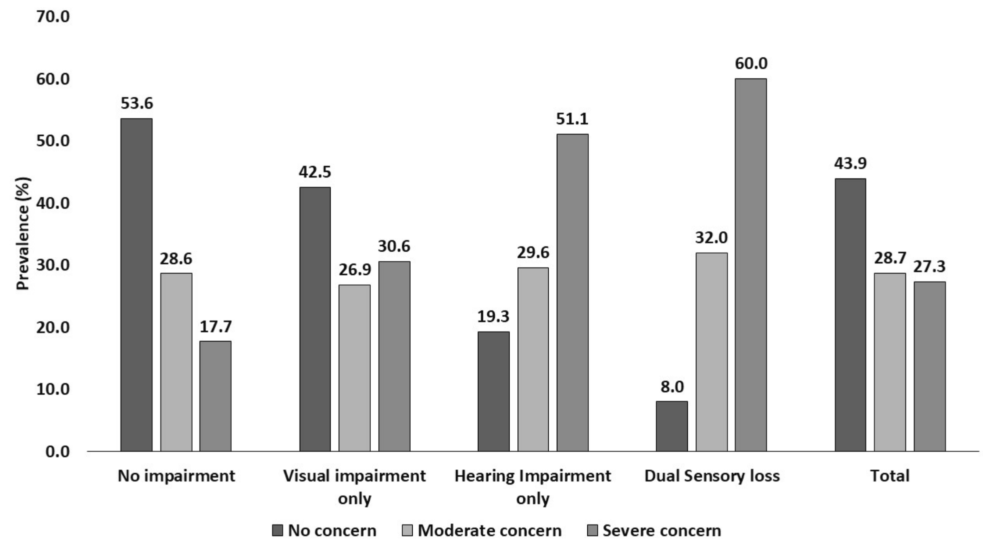

Sensory loss and fear of falling among elderly in residential care.

338x190mm (96 x 96 DPI)

STROBE 2007 (v4) Statement—Checklist of items that should be included in reports of *cross-sectional studies*

Section/Topic	Item #	Recommendation	Reported on page #
**Title and abstract**	1	(*a*) Indicate the study's design with a commonly used term in the title or the abstract	1
		(*b*) Provide in the abstract an informative and balanced summary of what was done and what was found	1
**Introduction**			
Background/rationale	2	Explain the scientific background and rationale for the investigation being reported	3
Objectives	3	State specific objectives, including any pre-specified hypotheses	3
**Methods**			
Study design	4	Present key elements of study design early in the paper	3
Setting	5	Describe the setting, locations, and relevant dates, including periods of recruitment, exposure, follow-up, and data collection	4,5
Participants	6	(*a*) Give the eligibility criteria, and the sources and methods of selection of participants	5,
Variables	7	Clearly define all outcomes, exposures, predictors, potential confounders, and effect modifiers. Give diagnostic criteria, if applicable	6,7
Data sources/ measurement	8*	For each variable of interest, give sources of data and details of methods of assessment (measurement). Describe comparability of assessment methods if there is more than one group	6,7
Bias	9	Describe any efforts to address potential sources of bias	NA
Study size	10	Explain how the study size was arrived at	5
Quantitative variables	11	Explain how quantitative variables were handled in the analyses. If applicable, describe which groupings were chosen and why	7
Statistical methods	12	(*a*) Describe all statistical methods, including those used to control for confounding	7
		(*b*) Describe any methods used to examine subgroups and interactions	7
		(*c*) Explain how missing data were addressed	NA
		(*d*) If applicable, describe analytical methods taking account of sampling strategy	NA
		(*e*) Describe any sensitivity analyses	NA
**Results**			

For peer review only

Participants	13*	(a) Report numbers of individuals at each stage of study—eg numbers potentially eligible, examined for eligibility, confirmed eligible, included in the study, completing follow-up, and analysed	NA
		(b) Give reasons for non-participation at each stage	NA
		(c) Consider use of a flow diagram	NA
Descriptive data	14*	(a) Give characteristics of study participants (eg demographic, clinical, social) and information on exposures and potential confounders	7
		(b) Indicate number of participants with missing data for each variable of interest	NA
Outcome data	15*	Report numbers of outcome events or summary measures	7
Main results	16	(*a*) Give unadjusted estimates and, if applicable, confounder-adjusted estimates and their precision (eg, 95% confidence interval). Make clear which confounders were adjusted for and why they were included	7
		(*b*) Report category boundaries when continuous variables were categorized	7
		(*c*) If relevant, consider translating estimates of relative risk into absolute risk for a meaningful time period	7
Other analyses	17	Report other analyses done—eg analyses of subgroups and interactions, and sensitivity analyses	7,8
**Discussion**			
Key results	18	Summarise key results with reference to study objectives	11,12
Limitations	19	Discuss limitations of the study, taking into account sources of potential bias or imprecision. Discuss both direction and magnitude of any potential bias	12,13
Interpretation	20	Give a cautious overall interpretation of results considering objectives, limitations, multiplicity of analyses, results from similar studies, and other relevant evidence	13
Generalisability	21	Discuss the generalisability (external validity) of the study results	13
**Other information**			
Funding	22	Give the source of funding and the role of the funders for the present study and, if applicable, for the original study on which the present article is based	14

*Give information separately for cases and controls in case-control studies and, if applicable, for exposed and unexposed groups in cohort and cross-sectional studies.

Note: An Explanation and Elaboration article discusses each checklist item and gives methodological background and published examples of transparent reporting. The STROBE checklist is best used in conjunction with this article (freely available on the Web sites of PLoS Medicine at http://www.plosmedicine.org/, Annals of Internal Medicine at http://www.annals.org/, and Epidemiology at http://www.epidem.com/). Information on the STROBE Initiative is available at www.strobe-statement.org.

For peer review only

A cross sectional study of prevalence and correlates of fear of falling in the older people in residential care in India – the Hyderabad Ocular Morbidity in Elderly Study

Journal:	*BMJ Open*
Manuscript ID	bmjopen-2023-080973.R2
Article Type:	Original research
Date Submitted by the Author:	23-Apr-2024

Complete List of Authors:	Marmamula, Srinivas; LV Prasad Eye Institute, GPR ICARE; University of New South Wales<br>Kumbham, Thirupathi Reddy; LV Prasad Eye Institute, GPR ICARE<br>Modepalli, Satya Brahmanandam ; LV Prasad Eye Institute, Gullapalli Pratibha Rao International Centre for Advancement of Rural Eye care<br>Barrenkala, Navya Rekha; LV Prasad Eye Institute<br>Keeffe, Jill; LV Prasad Eye Institute, GPR ICARE<br>Friedman, David; Massachusetts Eye and Ear Infirmary

**Primary Subject Heading**:	Geriatric medicine
Secondary Subject Heading:	Epidemiology, Ophthalmology, Public health
Keywords:	Aging, EPIDEMIOLOGY, GERIATRIC MEDICINE, PUBLIC HEALTH

SCHOLARONE™
Manuscripts

I, the Submitting Author has the right to grant and does grant on behalf of all authors of the Work (as defined in the below author licence), an exclusive licence and/or a non-exclusive licence for contributions from authors who are: i) UK Crown employees; ii) where BMJ has agreed a CC-BY licence shall apply, and/or iii) in accordance with the terms applicable for US Federal Government officers or employees acting as part of their official duties; on a worldwide, perpetual, irrevocable, royalty-free basis to BMJ Publishing Group Ltd ("BMJ") its licensees and where the relevant Journal is co-owned by BMJ to the co-owners of the Journal, to publish the Work in this journal and any other BMJ products and to exploit all rights, as set out in our licence.

The Submitting Author accepts and understands that any supply made under these terms is made by BMJ to the Submitting Author unless you are acting as an employee on behalf of your employer or a postgraduate student of an affiliated institution which is paying any applicable article publishing charge ("APC") for Open Access articles. Where the Submitting Author wishes to make the Work available on an Open Access basis (and intends to pay the relevant APC), the terms of reuse of such Open Access shall be governed by a Creative Commons licence – details of these licences and which Creative Commons licence will apply to this Work are set out in our licence referred to above.

Other than as permitted in any relevant BMJ Author's Self Archiving Policies, I confirm this Work has not been accepted for publication elsewhere, is not being considered for publication elsewhere and does not duplicate material already published. I confirm all authors consent to publication of this Work and authorise the granting of this licence.

For peer review only

A cross sectional study of prevalence and correlates of fear of falling in the older people in residential care in India – the Hyderabad Ocular Morbidity in Elderly Study

Srinivas Marmamula,[1,2,3,4,5*] Thirupathi Reddy K,[1,2] Satya Brahmanandam Modepalli, [1,2] Navya Rekha Barrenkala,[1] Jill E Keeffe,[1] David S Friedman[6]

[1] Allen Foster Community Eye Health Research Centre, Gullapalli Pratibha Rao International Centre for Advancement of Rural Eye care, L V Prasad Eye Institute, Hyderabad, India

[2] Brien Holden Institute of Optometry and Vision Science, L V Prasad Eye Institute, Hyderabad, India

[3] Wellcome Trust / Department of Biotechnology India Alliance, L V Prasad Eye Institute, Hyderabad, India

[4] School of Optometry and Vision Science, University of New South Wales, Sydney, Australia

[5] Harvard T.H. Chan School of Public Health, Boston, MA, USA

[6] Massachusetts Eye and Ear, Harvard Medical School, Harvard Medical School, Department of Ophthalmology, Boston, USA

Running Title: Fear of falling in the older people in India

***Corresponding author:** Dr. Srinivas Marmamula, Gullapalli Pratibha Rao International Centre for Advancement of Rural Eye care, L V Prasad Eye Institute, Hyderabad, India. 500034.

Email: sri.marmamula@lvpei.org

Total number of words: 3217 words excluding title page, abstract, acknowledgements, references, tables and figure legends

For peer review only - http://bmjopen.bmj.com/site/about/guidelines.xhtml

ABSTRACT

Objective: To report the prevalence and risk factors for the fear of falling (FOF) among older individuals living in residential care facilities in India.

Design: Cross sectional study

Setting: Homes for the aged centres in Hyderabad, India

Participants: The study included individuals aged ≥60 years from homes for the aged centres. The participants underwent a comprehensive eye examination in make-shift clinics setup in homes. Trained investigators collected the personal and demographic information of the participants using the Patient Health Questionnaire-9 and Hearing Handicap Inventory for Elderly questionnaire in the vernacular language. FOF was assessed using the Short Falls Efficacy Scale. The presence of hearing and vision impairment in the same individual was considered dual sensory impairment (DSI). A multiple logistic regression analysis was done to assess the factors associated with FOF.

Primary Outcome measure: Fear of Falling (FOF)

Results: In total, 867 participants were included from 41 homes for the aged centres in the analyses. The mean (±SD) age of the participants was 74.2 (±8.3) years (range: 60–96 years). The prevalence of FOF was 56.1% (95% CI:52.7–59.4; n=486). The multivariate analysis showed that those with DSI had eleven times higher odds of reporting FOF than those with no impairment (OR:11.14; 95% CI:3.15–41.4.) Similarly, those with moderate depression had seven times higher odds (OR:6.85; 95% CI:3.70–12.70), and those with severe depression had eight times higher odds (OR:8.13; 95% CI: 3.50–18.90) of reporting FOF. A history of falls in the last year was also associated with increased odds for FOF (OR:1.52; 95% CI:1.03–2.26).

Conclusion: FOF is common among older individuals in residential care in India. Depression, falling in the previous year, and DSI were strongly associated with FOF. A cross-disciplinary approach may be required to address FOF among older people in residential care in India.

Keywords: Fear of falling, dual sensory impairment, depression, older people

For peer review only

For peer review only - http://bmjopen.bmj.com/site/about/guidelines.xhtml

ARTICLE SUMMARY:

Strengths and limitations of the study

- Hyderabad Ocular Morbidity in Elderly Study (HOMES) is a cross-sectional study that included a large number of older people in residential care facilities in an urban region in India.
- All participants underwent comprehensive assessments including visual acuity measurement, hearing assessment, depression, mobility and other assessments.
- A Multiple logistic regression analysis including vision impairment, hearing impairment and dual sensory impairment as covariates was done to assess the independent predictors of fear of falling in the older population.
- The hearing assessment was based on a questionnaire, and this might have underestimated the prevalence of lower grades of hearing impairments.
- The results of this study can only be generalised to the older population in residential care settings in urban regions in India.

For peer review only

INTRODUCTION

The proportion of older people (people aged 60 years and older) in India's population is on the rise, and it is expected to reach 20% of the total population by 2050.[1] This demographic shift will dramatically change the nature of healthcare in India, and research on healthy ageing among the older population is needed. Such research can provide evidence for planning interventions to promote healthy and active ageing. Societal and lifestyle changes are sweeping through India, changing the living arrangements for the older population.[2, 3] Multi-generational households are giving way to nuclear families and small households.[2, 3] Under these changing circumstances, homes for the aged or residential care facilities are emerging and taking root rapidly.[2, 3]

Fear of falling (FOF) is a term used to describe an individual's loss of confidence in their ability to maintain balance, resulting in a worry about falling.[4] The prevalence of FOF in the older population ranges from 29–92% with a history of falls and 12–65% without a history of falls.[5] In India, FOF is a common challenge affecting over a third of the older population and affects their quality of life, physical well-being, and social functioning.[6-8] Moreover, it is associated with depression,[9] cognitive decline, and incidence of functional disability in the older population and can predict future falls.[10, 11] Several risk factors are associated with FOF, including vision impairment, hearing impairment, a history of falls, depression, and physical functioning.[12, 13] A recent longitudinal study concluded that FOF is as detrimental as previous falls in limiting the daily activities among the older people.[14]

Studies on active ageing, falls, and FOF in older people are more common in high-income countries.[12, 15] In India, such studies are still at a nascent stage, with very few studies reporting on FOF] and none reporting on the association of FOF between Dual Sensory Impairment (DSI, combined vision and hearing impairment) and depression among the older people in residential care.[6-8]

The Hyderabad Ocular Morbidity in Elderly Study (HOMES) is a large study conducted among older residents (aged $\geq$ 60 years) living in homes for the aged in Hyderabad, Telangana, India.[16] In addition to eye health, the study included an assessment of factors relevant to the health of older people, such as depression, falls, hearing impairment, and FOF.[16] Previous publications from this study reported on distance and near vision

For peer review only - http://bmjopen.bmj.com/site/about/guidelines.xhtml

impairment, dual sensory loss, depression, prevalence, and risk factors for falls in older people.[17-19] In this paper, we report on the prevalence and associations of FOF with vision loss, hearing loss, dual sensory loss, and depression among the older people living in homes for the aged.

MATERIAL AND METHODS

Ethics approval

The protocol for the HOMES study was approved by the Institutional Review Board of the Hyderabad Eye Research Foundation, L V Prasad Eye Institute, Hyderabad, India (Reference number: LEC-08-16-073). The study was performed in accordance with the Declaration of Helsinki. Each participant provided written informed consent. The data collection was carried out during 2017-2019.

Sampling method and recruitment of the participants

The FOF was assessed as a part of a larger eye health and visual impairment study.[16] A sample size of 916 participants was required based on an anticipated prevalence of 15% for avoidable visual impairment, a 20% precision, a 25% non-response rate, and a design effect of 1.4 to account for clustering for a cluster size of 40 people. Based on an anticipated prevalence of FOF of 33% as reported by a previous study, the sample required was 375 participants.[6] Of the 76 centres, 46 were selected (including five homes for the pilot) based on the proximity to a referral centre for eye care services and the willingness of the homes to participate in the study. Participants aged ≥60 years at the time of enumeration residing in the home for the aged for at least one month were included in the study.

Eye examination and other assessments

The examination procedures have been described in previous publications.[17-20] Briefly, monocular visual acuity (VA) was assessed at a three-meter distance under ambient illumination using a logMAR chart. The presenting and pinhole visual acuities were also assessed. Near vision was assessed using a logMAR chart at the standard distance of 40 cm. The anterior segment was examined using a portable slit lamp. A fundus examination and imaging were done for all participants and were graded by trained graders. Mobility was classified as independently mobile and mobile with assistance based on the participants' self-report and the interviewers' observations, including using a walking stick or assisted by other

For peer review only - http://bmjopen.bmj.com/site/about/guidelines.xhtml

people. The participants who were bedridden or in a wheelchair were not included in the study, though they were examined and provided with the necessary services.

Non-clinical questionnaires

Trained investigators conducted detailed interviews with the participants using structured questionnaires.[16, 20] All interviews were conducted before the eye examination. These included assessing the personal and demographic information (age, education level, marital status, and type of home) and systemic history (diabetes and hypertension). The Hindi Mini-Mental State Examination (HMSE) was used to assess cognitive status.[21] All the participants with an HMSE score of 20 or more were further interviewed and assessed for depression using the Patient Health Questionnaire-9 (PHQ-9).[22] The hearing was assessed using the Hearing Handicap Inventory for Elderly (HHIE-S) questionnaire, with a hearing aid, if any.[23] Questionnaires were administered in the vernacular languages (Telugu or Hindi).

FOF was assessed using the Short Falls Efficacy Scale (SFES - International) questionnaire.[24, 25] This questionnaire has seven questions on commonly performed tasks, including self-care, physical activity, and participation in social events. The response grades included Not at All Concerned, Somewhat Concerned, Fairly Concerned, and Very Concerned. All the response grades were added to get a cumulative FOF score.[24] The history of previous falls was documented by asking, “Have you ever fallen on the floor in the last one year?” in the vernacular language (Telugu or Hindi). The response was recorded as 0=No, 1=Yes, and 3= Cannot remember.[18] The participants also asked if they had a fall in the last two weeks.

Definitions

a. Fear of Falling (FOF): A cumulative score ≥9 was considered as an individual with FOF. It was used as a dichotomous variable to define FOF. It was also graded as low (≤8), moderate (9-13), and high concern (14-28).
b. Vision Impairment (VI): Presenting distance visual acuity (VA) worse than 6/18 in the better eye, without hearing impairment (HI).
c. Hearing impairment (HI): It was defined as an HHIE Score <10 on the HHIE-S, without visual impairment (VI).

For peer review only - http://bmjopen.bmj.com/site/about/guidelines.xhtml

d. Dual Sensory Impairment (DSI): The presence of both VI and HI in an individual was considered DSI.

e. Depression: An individual with a cumulative score of ≥10 on the PHQ-9 was considered as having depression. It was graded as none/mild (0-9), moderate (10-14), and moderate-severe/severe (14-17).

f. Multimorbidity: It was defined as reporting two or more non-communicable diseases (NCDs) in an individual.

g. Polypharmacy: It was defined as the use of five or more medications by an individual on a daily basis.

Patient and public involvement

The patients and the public were not involved in the design and conduct of the study.

Data management

Data analysis was conducted using Stata 14.0 (Stata Corp LP, College Station, TX).[26] For descriptive statistics, continuous variables were analysed using the Student's t-test, and the categorical variables were analysed using the chi-square test. The prevalence of FOF was estimated and presented with 95% confidence intervals (CI). In the multiple logistic regression analysis, FOF was used as a dichotomous outcome variable, and its association with personal and sociodemographic variables (age, gender and education), body mass index, multimorbidity, polypharmacy, depression, HI, VI, and DSI was evaluated. The variables were entered into the model one at a time, and the selection of covariates for the model was based on previous studies reporting on FOF.[27] The Hosmer-Lemeshow goodness of fit test was used to assess the model fit. Variance inflation factors were used to test for collinearity between the covariates after fitting a multiple regression model. The adjusted odds ratio (OR) with 95% confidence intervals (CI) were presented. Statistical significance was set at $p<0.05$ (two-tailed), and the exact p-values are reported.

RESULTS

Characteristics of the study participants

The personal and demographic characteristics of the participants have been described in previous publications.[19] The HOMES study included 1182 participants; this included 271 participants who were bedridden and 98 participants with HMSE scores worse than 20.

For peer review only - http://bmjopen.bmj.com/site/about/guidelines.xhtml

Of the remaining 867 participants included in the analyses, 537 (61.6%) were women, 116 (13.4%) had no education, 518 (59.8%) reported hypertension, and 263 (30.3%) reported diabetes. The mean (±SD) age of the participants was 74.2 (±8.3) years (range: 60–96 years). In terms of mobility status, 613 (70.7%) were independent, and the remaining 254 (29.3%) needed assistance from others or used walking aids for mobility. In total, 363 (41.9%) participants had multimorbidity and 161 (18.6%) reported polypharmacy.

Sensory impairments

In total, 548 (63.2%) participants had no sensory loss, 134 (15.5%) had VI, 135 (15.6%) had HI, and 50 (5.8%) had DSI. Among those with VI, the causes of VI were cataract (44.0%; n=59), uncorrected refractive errors (35.5%; n=48), posterior capsular opacification (9%; n=12), and others (11%; n=15).

Prevalence of FOF

The prevalence of FOF (moderate + high concern) was 56.1% (95% CI: 52.7–59.4; n=486). In total, 381 (43.9%) participants reported no concern about falling, 249 (28.7%) had a moderate concern, and 237 (27.3%) reported a high concern. The prevalence of FOF varied with the nature of sensory loss, with the highest prevalence among those with DSI (92.0%), followed by HI (80.1%) and VI (57.5%). The prevalence of FOF among those with a history of falls was 69.4% (95% CI: 63.2–75.2) compared to 50.9% (95% CI: 46.9–54.9) among those without a history of falling. Those with multimorbidity had a significantly higher prevalence of FOF (Table 1).

Table 1: Personal, demographic, and health-related characteristics and prevalence of fear of falling (n=867) among older population in homes for the aged centres.

	Total in the sample	Fear of Falling † n (row %)	Fear of Falling - Prevalence (95% CI)
**Age group (years)**			
60-69	263	118 (44.9)	44. 9 (38.7–51.0)
70-79	345	186 (53.9)	53.9 (48.5–59.3)
80 and above	259	182 (70.3)	70.3 (64.3–75.8)
**Gender**			
Male	330	171 (51.8)	51.8 (46.3–57.3)
Female	537	315 (58.7)	58.7 (54.4–62.9))
**Education level**			
Any education	751	400 (53.3)	53.3 (49.6–56.9)
No education	116	86 (74.1)	74.1 (65.2–81.8)

For peer review only - http://bmjopen.bmj.com/site/about/guidelines.xhtml

**Body Mass Index**			
Underweight/Normal (≤24.9)	397	210 (52.9)	52.9 (47.8–57.9)
Overweight (25.0 – 29.9)	289	159 (55.0)	55.0 (49.1–60.8)
Obese (30 and above)	153	97 (63.4)	63.4 (55.2–71.0)
**Hypertension**			
No	349	157 (45.0)	45.0 (39.7–50.4)
Yes	518	329 (63.5)	63.5 (59.2-67.7)
**Diabetes**			
No	604	345 (57.1)	57.1 (53.1–61.1)
Yes	263	141 (53.6)	53.6 (47.4–59.7)
**Mobility status**			
Independently mobile	613	275 (44.9)	44.9 (40.9–49.9)
Mobility with support	254	211 (83.1)	83.1 (77.9–87.5)
**Depression**			
Mild/None	672	313 (46.6)	46.5 (42.7–50.4)
Moderate	104	89 (85.6)	85.6 (77.3–91.7)
Severe	91	84 (92.3)	92.3 (84.8–96.8)
**History of fall in a year**			
No fall	625	318 (50.9)	50.9 (46.9–54.9)
Falls reported	242	168 (69.4)	69.4 (63.2–75.2)
**Visual impairment only**			
No	733	409 (55.8)	55.8 (52.1–59.4)
Yes	134	77 (57.5)	57.4 (48.6–66.0)
**Hearing impairment only**			
No	732	377 (51.5)	51.5 (47.8–55.2)
Yes	135	109 (80.1)	80.1 (73.1–87.0)
**Dual Sensory Impairment**			
No	817	440 (53.9)	53.9 (50.4–57.3)
Yes	50	46 (92.0)	92.0 (80.8–97.8)
**Multimorbidity**			
No	504	261	51.7 (47.3–56.2)
Yes	363	225	62.0 (56.8–67.0)
**Polypharmacy**			
No	706	382	54.1 (50.3–57.8)
Yes	161	104	64.6 (56.7–72.0)
**Total**	**867**	**486 (56.1)**	**56.1 (52.7–59.4)**

†Fear of falling was defined as a score of >= 9 on the Short Falls Efficacy Scale (SFES - International) questionnaire; ‡BMI data not available on 28 participants.

Figure 1 shows the grades of FOF among the participants without any impairment, HI, VI, and DSI. The proportion of participants reporting a serious concern of falling was highest among those with DSI, followed by HI and VI (p<0.001).

For peer review only - http://bmjopen.bmj.com/site/about/guidelines.xhtml

Figure 1: Sensory loss and fear of falling among older people in residential care.

Risk factors for FOF

Table 2 shows the association between FOF and various risk factors. FOF was significantly associated with older age, high BMI, and lack of education. The older participants with dual sensory impairment had eleven times higher odds of FOF (OR: 11.43; 95% CI: 3.15–41.41, Table 2) than those with no sensory impairment. HI was significantly associated with FOF (OR: 3.34; 95% CI: 1.96–5.72); however, VI (OR: 1.51; 95% CI: 0.95–2.40) was not significantly (p=0.08) associated. Those with moderate depression had seven times (OR: 6.86; 95% CI: 3.70–12.70) higher odds of FOF, and those with severe depression had eight times higher odds (OR:8.13; 95% CI: 3.50–18.88). A history of falls over the past year was also associated with an increase in FOF (OR: 1.53; 95% CI: 1.03–2.26). Those needing assistance for mobility had five times higher odds of FOF (OR: 4.84; 95% CI: 3.1–7.44) compared to those with independent mobility. Polypharmacy and multimorbidity were not associated with FOF.

Table 2: Multiple logistic regression analysis, assessing the factors associated with fear of falling in older people living in residential care.(n=839)

	**Adjusted Odds Ratio (95% CI)**	**p-value**
**Age group (years)**		
60-69	Reference	
70-79	1.27 (0.86–1.88)	0.233
80 and above	1.84 (1.16–2.92)	0.010
**Gender**		
Male	Reference	
Female	1.35 (0.95–1.94)	0.097
**Education**		
Any education	Reference	
No education	2.31 (1.34–3.99)	0.003
**Body Mass Index (BMI)**		
Normal	Reference	
Overweight	1.28 (0.87–1.87)	0.215
Obese	2.03 (1.26–3.27)	0.003
**Multimorbidity**		
No	Reference	
Yes	1.22 (0.85–1.78)	0.273
**Polypharmacy**		
No	Reference	
Yes	1.36 (0.85–2.17)	0.193
**Mobility status**		

For peer review only - http://bmjopen.bmj.com/site/about/guidelines.xhtml

Independent mobility	Reference	
Mobility with support/aid	4.84 (3.15–7.44)	<0.001
**Depression**		
None	Reference	
Moderate	6.86 (3.71–12.70)	<0.001
Severe	8.13 (3.50–18.88)	<0.001
**History of fall in last year**		
No	Reference	
Yes	1.53 (1.03–2.26)	0.033
**Dual Sensory Impairment**		
No	Reference	
Yes	11.43 (3.15–41.42)	<0.001
**Vision impairment only**		
No	Reference	
Yes	1.51 (0.95–2.40)	0.084
**Hearing impairment only**		
No	Reference	
Yes	3.35 (1.96–5.72)	<0.001

DISCUSSION

Over half of the older people in residential care facilities had a fear of falling. In addition, nearly 90% of those with hearing and vision impairment (DSI) reported FOF. Earlier studies done in India reported a lower prevalence of FOF compared to the present study.[6-8] The possible reasons for a higher prevalence in this study could be explained by the differences in the living conditions of the older residents. Earlier studies reported on the older population living in a community setting, whereas those in the present study live in an institutional setting. Reports from other countries have shown a higher prevalence of FOF among residents in institutions.[28] No previous studies have reported FOF among older residents in institutional care in India. One study reported 42% prevalence of FOF among the older people in a hospital setting in India.[29]

Studies from other parts of the world have reported a higher prevalence of FOF among the older people living in institutional settings than those living in communities.[28, 30, 31] However, the results from studies done in high-income countries cannot be extrapolated to India. Nursing homes and homes for the aged in high-income countries are well-established, with clear protocols in terms of infrastructure requirements, dedicated personnel specialised in care for older people, and laws governing the quality of care. Homes for the aged in India are relatively new, and there are no standard minimum requirements for establishing such

For peer review only - http://bmjopen.bmj.com/site/about/guidelines.xhtml

institutions.[32] The facilities, personnel, and scope of service vary greatly based on the paying status of residents and the leadership of the facilities.[32] Homes in India are largely unmonitored and are run by private and non-government entities.[32] Due to these differences, the lifestyles of the older residents in homes in India and other high-income countries are not comparable.

The association between VI and FOF has been inconsistent across studies.[33-35] Some have reported a positive association,[36] while others have found no associations.[35, 37, 38] There was no association between VI and FOF in this study after adjusting for other covariates. A possible reason for the inconsistent associations across the studies is the differences in the definition used for VI. However, there could also be a small association, which most studies are underpowered to detect. We did find a positive association, but it was not statistically significant. It is also possible that the studies that reported an association between VI and FOF could have included those with DSI, which may have increased the association, as people with DSI were more likely to report FOF. Those with Dual Sensory Impairment (DSI) had a substantially higher FOF compared to those with single sensory loss, either VI or HI. A recent study on the older population in nursing homes in India also reported a higher prevalence of FOF among those with dual sensory loss.[28]

The studies on eye health do not include the assessment of hearing as a routine procedure. We found that approximately 90% of those with DSI reported FOF, highlighting the need to assess both hearing status and eye health in the older population for adequate care and when carrying out research studies. Finally, though earlier studies reported a higher prevalence of FOF among women, there was no significant association with gender in this study.[39] FOF is associated independently with vision impairment and hearing impairment.[34] A recent study reported a higher prevalence of FOF among nursing home residents with sensory loss, as over half of them had FOF.[28] A study by Murphy et al. reported that older people who had restricted their daily activities out of fear of falling had a higher burden of depressive symptoms than those who only reported fear of falling.[40] A meta-analysis reported that untreated depression is significantly associated with an increased risk of falling, which in turn may lead to FOF.[27]

FOF was associated with depression in this study, which is consistent with other studies.[13, 30, 36, 41-46] Causality is impossible to demonstrate; however, several pathways for FOF

For peer review only - http://bmjopen.bmj.com/site/about/guidelines.xhtml

leading to depression could be hypothesized. FOF is associated with reduced physical activity in older people.[47] This is evident in the current study by a higher odds for FOF among those needing assistance for mobility. The reduced activity results in a more sedentary lifestyle, increasing social isolation and leading to depression. However, it is also possible that those with higher levels of depression tend to become sedentary and limit their social interaction and mobility.

In our previous, we reported that those with DSI had ten times higher odds to have depression compared to those with single sensory loss.[19] In this study, hearing impairment was independently associated with FOF after adjusting for other covariates. The possible explanation for this finding could be the changes in gait balance due to hearing impairment, leading to an increased FOF. A greater FOF was reported among those with lower gait velocity.[48]

Independent mobility is a measure of physical function, and a loss of it is associated with falls and FOF.[49-51] In this study, those requiring assistance for their mobility were five times more likely to have FOF compared to those who had independent mobility. Poor physical function may result in a loss of confidence among older people, resulting in an increased FOF.[50] In a longitudinal study, FOF was associated with a lack of independent mobility and poor physical performance at the end of two years.[14] This indicates the need to address FOF in the older population to prevent a decline in physical functions.

The association between falls and FOF is reported in a few studies in India.[7, 37] FOF was higher among those with a history of falls in India.[7, 33] Adverse consequences of falls in the older people are also well-known, which in itself may lead to FOF. Fear itself is an innate response that has an evolutionary importance and is a protective instinct. Similarly, a fear of falling could act as an instinctual defence mechanism to prevent falls, but only to a certain threshold.[31, 52] Beyond this threshold, FOF could impair physical functioning and negatively impact the quality of life in older people.[31, 52] A recent systematic review has concluded that FOF adversely impacts the quality of life of older residents in community settings and institutional care.[53]

Several interventions have been reported to address FOF in older people living in communities and nursing homes. These interventions include cognitive and behavioural

For peer review only - http://bmjopen.bmj.com/site/about/guidelines.xhtml

interventions, occupational therapy, and physical exercises, including balance training, yoga, and tai chi.[43, 54-60] In addition to addressing FOF, these interventions may also have a resonating effect, alleviating depression, leading to better quality of life, and promoting well-being in the older population.[61-63] However, studies on the impact of such interventions that address FOF are limited in India. The older residents living in institutional care present a unique opportunity where interventions can be applied to more individuals in one setting, even with limited resources, than the older population diffused across communities. Studies also indicate an association between FOF and cognitive decline,[36, 64] which underpins the need to address FOF among older people.

This study benefits from a large cohort of older participants and a comprehensive assessment (inclusion of dual sensory impairment and depression). Some limitations include our decision to exclude participants with severe mobility issues and those with cognitive impairment, as indicated by the lower HSME scores. This may have led to an underestimation of the prevalence of FOF in our study. However, it is unlikely to have led to an inaccurate assessment of associations. Environmental and infrastructure factors also influence FOF, which were not evaluated in our study. A direct extrapolation of our results to those who live in the communities is limited due to the differences in the types of residences and the activities of people. Moreover, while visual acuity assessment using the standard procedures used in the clinic, hearing was assessed using a questionnaire, which is not an objective assessment. This limitation could have led to an underestimation of the prevalence of HI in our study. HOMES was an observational study hence causality cannot be established.

In conclusion, FOF is common among the older population in residential care in India. It is especially common among those with depression and dual sensory impairment. With the increase in the older population and the rapid increase in the number of homes for the aged in urban areas, these findings are relevant for policy and healthcare service planning for older people. The findings of this study can help inform planners to develop appropriate interventions to address FOF in the older population and, promote healthy and active ageing among these residents.

Acknowledgments: The authors thank the individuals for their participation in the study. Mr. Shashank Yellapragada, Rajesh Challa, Madhuri Bakki is acknowledged for their assistance in data collection. Ratnakar Yellapragada and Ms. Muni Rajya Lakshmi are acknowledged

For peer review only - http://bmjopen.bmj.com/site/about/guidelines.xhtml

For peer review only

for her support with data management. The authors also thank Mr. Abhinav Sekar for his language inputs on earlier versions of the manuscript.

Competing interests: "We have read and understood BMJ policy on declaration of interests and declare that we have no competing interests."

Funding Support: This work was supported by Wellcome Trust/DBT India Alliance Fellowship [IA/CPHE/14/1/501506] awarded to Dr. Srinivas Marmamula and Hyderabad Eye Research Foundation (HERF), India.

Contributorship Statement: SM conceived the idea, designed and conducted the study, analyzed the data and wrote the manuscript. TRK, SBM and NRB were involved in data collection and data management and provided intellectual inputs on the earlier versions of the manuscript. JEK and DSF critically reviewed the manuscript and provided intellectual inputs. SM bears primary responsibility for the final content of the manuscript and the accuracy of the data. SM, TRK, SBM, NRB, JEK and DSF have approved the final manuscript.

Data sharing statement: No additional data are available.

For peer review only

REFERENCES

1. UN. World Population Prospects 2017 United Nations, Department of Economic and Social Affairs, Population Division (2017, 2017.
2. Agrawal S. Effect of living arrangements on health status of elderly in India. Asian Population Studies 2012;**8**(1):87-101 doi: 10.1080/17441730.2012.646842[published Online First: Epub Date]|.
3. Mane AB. Ageing in India: some social challenges to elderly care. J Gerontol Geriatr Res 2016;**5**(2):e136
4. Legters K. Fear of falling. Phys Ther 2002;**82**(3):264-72
5. Malini FM, Lourenco RA, Lopes CS. Prevalence of fear of falling in older adults, and its associations with clinical, functional and psychosocial factors: the Frailty in Brazilian Older People-Rio de Janeiro study. Geriatr Gerontol Int 2016;**16**(3):336-44 doi: 10.1111/ggi.12477[published Online First: Epub Date]|.
6. Mane AB, Sanjana T, Patil PR, Sriniwas T. Prevalence and correlates of fear of falling among elderly population in urban area of Karnataka, India. Journal of mid-life health 2014;**5**(3):150-5 doi: 10.4103/0976-7800.141224[published Online First: Epub Date]|.
7. Pitchai P, Dedhia HB, Bhandari N, Krishnan D, D'Souza NRJ, Bellara JM. Prevalence, risk factors, circumstances for falls and level of functional independence among geriatric population - A descriptive study. Indian journal of public health 2019;**63**(1):21-26 doi: 10.4103/ijph.IJPH_332_17[published Online First: Epub Date]|.

For peer review only - http://bmjopen.bmj.com/site/about/guidelines.xhtml

8. Dhavale S, Singh S, Parasher RK. Factors Associated with Fear of Falls in Indian Elderly: A Systematic Review. Indian Journal of Public Health Research & Development 2019;**10**(10)
9. Dukyoo J, Juhee L, Lee SM. A meta-analysis of fear of falling treatment programs for the elderly. West J Nurs Res 2009;**31**(1):6-16 doi: 10.1177/0193945908320466[published Online First: Epub Date]|.
10. Shirooka H, Nishiguchi S, Fukutani N, et al. Cognitive impairment is associated with the absence of fear of falling in community-dwelling frail older adults. Geriatr Gerontol Int 2017;**17**(2):232-38 doi: 10.1111/ggi.12702[published Online First: Epub Date]|.
11. Auais M, French S, Alvarado B, Pirkle C, Belanger E, Guralnik J. Fear of Falling Predicts Incidence of Functional Disability 2 Years Later: A Perspective From an International Cohort Study. The journals of gerontology. Series A, Biological sciences and medical sciences 2018;**73**(9):1212-15 doi: 10.1093/gerona/glx237[published Online First: Epub Date]|.
12. Jung D. Fear of falling in older adults: comprehensive review. Asian Nurs Res (Korean Soc Nurs Sci) 2008;**2**(4):214-22 doi: 10.1016/S1976-1317(09)60003-7[published Online First: Epub Date]|.
13. Mishra N, Mishra AK, Bidija M. A study on correlation between depression, fear of fall and quality of life in elderly individuals. Int J Res Med Sci 2017;**5**(4):1456
14. Liu M, Hou T, Li Y, et al. Fear of falling is as important as multiple previous falls in terms of limiting daily activities: a longitudinal study. BMC Geriatr 2021;**21**(1):350 doi: 10.1186/s12877-021-02305-8[published Online First: Epub Date]|.
15. Scheffer AC, Schuurmans MJ, van Dijk N, van der Hooft T, de Rooij SE. Fear of falling: measurement strategy, prevalence, risk factors and consequences among older persons. Age and ageing 2008;**37**(1):19-24 doi: 10.1093/ageing/afm169[published Online First: Epub Date]|.
16. Marmamula S, Barrenkala NR, Challa R, et al. Hyderabad Ocular Morbidity in Elderly Study (HOMES) - Rationale, Study Design and Methodology. Ophthalmic Epidemiol 2020;**27**(1):83-92 doi: 10.1080/09286586.2019.1683867[published Online First: Epub Date]|.
17. Marmamula S, Barrenakala NR, Challa R, et al. Prevalence and risk factors for visual impairment among elderly residents in 'homes for the aged' in India: the Hyderabad Ocular Morbidity in Elderly Study (HOMES). Br J Ophthalmol 2021;**105**(1):32-36 doi: 10.1136/bjophthalmol-2019-315678[published Online First: Epub Date]|.
18. Marmamula S, Barrenkala NR, Challa R, et al. Falls and visual impairment among elderly residents in 'homes for the aged' in India. Sci Rep 2020;**10**(1):13389 doi: 10.1038/s41598-020-70066-2[published Online First: Epub Date]|.
19. Marmamula S, Kumbham TR, Modepalli SB, Barrenkala NR, Yellapragada R, Shidhaye R. Depression, combined visual and hearing impairment (dual sensory impairment): a hidden multi-morbidity among the elderly in Residential Care in India. Sci Rep 2021;**11**(1):16189 doi: 10.1038/s41598-021-95576-5[published Online First: Epub Date]|.
20. Marmamula S, Barrenkala NR, Rajesh C, et al. Hyderabad Ocular Morbidity in Elderly Study (HOMES) – Rationale, Study design and Methodology. Ophthalmic Epidemiol 2019((In press)) doi: 10.1080/09286586.2019.1683867[published Online First: Epub Date]|.
21. Folstein MF, Folstein SE, McHugh PR. "Mini-mental state". A practical method for grading the cognitive state of patients for the clinician. J Psychiatr Res 1975;**12**(3):189-98

For peer review only - http://bmjopen.bmj.com/site/about/guidelines.xhtml

22. Kroenke K, Spitzer RL, Williams JB, Lowe B. The Patient Health Questionnaire Somatic, Anxiety, and Depressive Symptom Scales: a systematic review. Gen Hosp Psychiatry 2010;**32**(4):345-59 doi: 10.1016/j.genhosppsych.2010.03.006[published Online First: Epub Date]|.
23. Ventry IM, Weinstein BE. The hearing handicap inventory for the elderly: a new tool. Ear Hear 1982;**3**(3):128-34
24. Kempen GI, Yardley L, van Haastregt JC, et al. The Short FES-I: a shortened version of the falls efficacy scale-international to assess fear of falling. Age and ageing 2008;**37**(1):45-50 doi: 10.1093/ageing/afm157[published Online First: Epub Date]|.
25. Tinetti ME, Richman D, Powell L. Falls efficacy as a measure of fear of falling. Journal of gerontology 1990;**45**(6):P239-P43
26. StataCorp. Stata Statistical Software: Release 14. College Station, TX: StataCorp LP., 2014.
27. Deandrea S, Lucenteforte E, Bravi F, Foschi R, La Vecchia C, Negri E. Risk factors for falls in community-dwelling older people: a systematic review and meta-analysis. Epidemiology 2010;**21**(5):658-68 doi: 10.1097/EDE.0b013e3181e89905[published Online First: Epub Date]|.
28. Lach HW, Lozano AJ, Hanlon AL, Cacchione PZ. Fear of falling in sensory impaired nursing home residents. Aging Ment Health 2020;**24**(3):474-80 doi: 10.1080/13607863.2018.1537359[published Online First: Epub Date]|.
29. Dhar M, Kaeley N, Mahala P, Saxena V, Pathania M. The Prevalence and Associated Risk Factors of Fear of Fall in the Elderly: A Hospital-Based, Cross-Sectional Study. Cureus 2022;**14**(3):e23479 doi: 10.7759/cureus.23479[published Online First: Epub Date]|.
30. Chou K-L, Yeung F, Wong E. Fear of falling and depressive symptoms in Chinese elderly living in nursing homes: fall efficacy and activity level as mediator or moderator? Aging & mental health 2005;**9**(3):255-61
31. Gillespie SM, Friedman SM. Fear of falling in new long-term care enrollees. Journal of the American Medical Directors Association 2007;**8**(5):307-13
32. Liebig PS. Old-age homes and services: old and new approaches to aged care. Journal of aging & social policy 2003;**15**(2-3):159-78 doi: 10.1300/J031v15n02_10[published Online First: Epub Date]|.
33. Subramanian MS, Singh V, Chatterjee P, Dwivedi SN, Dey AB. Prevalence and predictors of falls in a health-seeking older population: An outpatient-based study. Aging Med (Milton) 2020;**3**(1):25-31 doi: 10.1002/agm2.12096[published Online First: Epub Date]|.
34. White UE, Black AA, Wood JM, Delbaere K. Fear of falling in vision impairment. Optom Vis Sci 2015;**92**(6):730-5 doi: 10.1097/OPX.0000000000000596[published Online First: Epub Date]|.
35. Donoghue OA, Ryan H, Duggan E, et al. Relationship between fear of falling and mobility varies with visual function among older adults. Geriatr Gerontol Int 2014;**14**(4):827-36 doi: 10.1111/ggi.12174[published Online First: Epub Date]|.
36. Vo THM, Nakamura K, Seino K, Nguyen HTL, Van Vo T. Fear of falling and cognitive impairment in elderly with different social support levels: findings from a community survey in Central Vietnam. BMC Geriatr 2020;**20**(1):141 doi: 10.1186/s12877-020-01533-8[published Online First: Epub Date]|.
37. Friedman SM, Munoz B, West SK, Rubin GS, Fried LP. Falls and fear of falling: which comes first? A longitudinal prediction model suggests strategies for primary and secondary prevention. Journal of the American Geriatrics Society 2002;**50**(8):1329-35

For peer review only

For peer review only - http://bmjopen.bmj.com/site/about/guidelines.xhtml

38. Deshpande N, Metter EJ, Lauretani F, Bandinelli S, Guralnik J, Ferrucci L. Activity restriction induced by fear of falling and objective and subjective measures of physical function: a prospective cohort study. Journal of the American Geriatrics Society 2008;**56**(4):615-20 doi: 10.1111/j.1532-5415.2007.01639.x[published Online First: Epub Date]|.
39. Gazibara T, Kurtagic I, Kisic-Tepavcevic D, et al. Falls, risk factors and fear of falling among persons older than 65 years of age. Psychogeriatrics : the official journal of the Japanese Psychogeriatric Society 2017;**17**(4):215-23 doi: 10.1111/psyg.12217[published Online First: Epub Date]|.
40. Murphy SL, Williams CS, Gill TM. Characteristics associated with fear of falling and activity restriction in community-living older persons. Journal of the American Geriatrics Society 2002;**50**(3):516-20 doi: 10.1046/j.1532-5415.2002.50119.x[published Online First: Epub Date]|.
41. van Haastregt JC, Zijlstra GR, van Rossum E, van Eijk JTM, Kempen GI. Feelings of anxiety and symptoms of depression in community-living older persons who avoid activity for fear of falling. The American Journal of Geriatric Psychiatry 2008;**16**(3):186-93
42. Cumming RG, Salkeld G, Thomas M, Szonyi G. Prospective study of the impact of fear of falling on activities of daily living, SF-36 scores, and nursing home admission. The Journals of Gerontology Series A: Biological Sciences and Medical Sciences 2000;**55**(5):M299-M305
43. Huang TT, Chung ML, Chen FR, Chin YF, Wang BH. Evaluation of a combined cognitive-behavioural and exercise intervention to manage fear of falling among elderly residents in nursing homes. Aging Ment Health 2016;**20**(1):2-12 doi: 10.1080/13607863.2015.1020411[published Online First: Epub Date]|.
44. Berkman LF, Sekher T, Capistrant B, Zheng Y. Social networks, family, and care giving among older adults in India. Aging in Asia: Findings from new and emerging data initiatives: National academies Press (US), 2012.
45. Singh L, Singh PK, Arokiasamy P. Social Network and Mental Health Among Older Adults in Rural Uttar Pradesh, India: A Cross-Sectional Study. J Cross Cult Gerontol 2016;**31**(2):173-92 doi: 10.1007/s10823-016-9286-0[published Online First: Epub Date]|.
46. Hajek A, Bock JO, Konig HH. Psychological correlates of fear of falling: Findings from the German Aging Survey. Geriatr Gerontol Int 2018;**18**(3):396-406 doi: 10.1111/ggi.13190[published Online First: Epub Date]|.
47. Dsouza SA, Rajashekar B, Dsouza H, Kumar K. Falls in Indian older adults: a barrier to active ageing. Asian J Gerontol Geriatr 2014;**9**(1):1-8
48. Reelick MF, van Iersel MB, Kessels RP, Rikkert MG. The influence of fear of falling on gait and balance in older people. Age and ageing 2009;**38**(4):435-40 doi: 10.1093/ageing/afp066[published Online First: Epub Date]|.
49. Ayoubi F, Launay C, Annweiler C, Fantino B, Kabeshova A, Beauchet O. Fear of falling, falls, and gait variability in older community-dwelling individuals: is there an association? Journal of the American Geriatrics Society 2013;**61**(7):1236-8 doi: 10.1111/jgs.12350[published Online First: Epub Date]|.
50. Brouwer B, Musselman K, Culham E. Physical function and health status among seniors with and without a fear of falling. Gerontology 2004;**50**(3):135-41
51. Jefferis BJ, Iliffe S, Kendrick D, et al. How are falls and fear of falling associated with objectively measured physical activity in a cohort of community-dwelling older men? BMC Geriatr 2014;**14**:114 doi: 10.1186/1471-2318-14-114[published Online First: Epub Date]|.

For peer review only - http://bmjopen.bmj.com/site/about/guidelines.xhtml

52. Murphy SL, Dubin JA, Gill TM. The development of fear of falling among community-living older women: predisposing factors and subsequent fall events. The Journals of Gerontology Series A: Biological Sciences and Medical Sciences 2003;**58**(10):M943-M47
53. Schoene D, Heller C, Aung YN, Sieber CC, Kemmler W, Freiberger E. A systematic review on the influence of fear of falling on quality of life in older people: is there a role for falls? Clinical interventions in aging 2019;**14**:701-19 doi: 10.2147/CIA.S197857[published Online First: Epub Date]|.
54. Tennstedt S, Howland J, Lachman M, Peterson E, Kasten L, Jette A. A randomized, controlled trial of a group intervention to reduce fear of falling and associated activity restriction in older adults. The Journals of Gerontology Series B: Psychological Sciences and Social Sciences 1998;**53**(6):P384-P92
55. Walker JE, Howland J. Falls and fear of falling among elderly persons living in the community: occupational therapy interventions. Am J Occup Ther 1991;**45**(2):119-22 doi: 10.5014/ajot.45.2.119[published Online First: Epub Date]|.
56. Zhang J-G, Ishikawa-Takata K, Yamazaki H, Morita T, Ohta T. The effects of Tai Chi Chuan on physiological function and fear of falling in the less robust elderly: an intervention study for preventing falls. Archives of gerontology and geriatrics 2006;**42**(2):107-16
57. Sattin RW, Easley KA, Wolf SL, Chen Y, Kutner MH. Reduction in fear of falling through intense tai chi exercise training in older, transitionally frail adults. Journal of the American Geriatrics Society 2005;**53**(7):1168-78
58. Liu TW, Ng GYF, Ng SSM. Effectiveness of a combination of cognitive behavioral therapy and task-oriented balance training in reducing the fear of falling in patients with chronic stroke: study protocol for a randomized controlled trial. Trials 2018;**19**(1):168 doi: 10.1186/s13063-018-2549-z[published Online First: Epub Date]|.
59. Jung D, Lee J, Lee S-M. A meta-analysis of fear of falling treatment programs for the elderly. Western journal of nursing research 2009;**31**(1):6-16
60. Sjosten N, Vaapio S, Kivela SL. The effects of fall prevention trials on depressive symptoms and fear of falling among the aged: a systematic review. Aging Ment Health 2008;**12**(1):30-46 doi: 10.1080/13607860701366079[published Online First: Epub Date]|.
61. Chobe S, Chobe M, Metri K, Patra SK, Nagaratna R. Impact of Yoga on cognition and mental health among elderly: A systematic review. Complementary therapies in medicine 2020;**52**:102421 doi: 10.1016/j.ctim.2020.102421[published Online First: Epub Date]|.
62. Cramer H, Lauche R, Langhorst J, Dobos G. Yoga for depression: a systematic review and meta-analysis. Depress Anxiety 2013;**30**(11):1068-83 doi: 10.1002/da.22166[published Online First: Epub Date]|.
63. Zettergren KK, Lubeski JM, Viverito JM. Effects of a yoga program on postural control, mobility, and gait speed in community-living older adults: a pilot study. J Geriatr Phys Ther 2011;**34**(2):88-94 doi: 10.1519/JPT.0b013e31820aab53[published Online First: Epub Date]|.
64. Noh HM, Roh YK, Song HJ, Park YS. Severe Fear of Falling Is Associated With Cognitive Decline in Older Adults: A 3-Year Prospective Study. J Am Med Dir Assoc 2019;**20**(12):1540-47 doi: 10.1016/j.jamda.2019.06.008[published Online First: Epub Date]|.

For peer review only - http://bmjopen.bmj.com/site/about/guidelines.xhtml

For peer review only

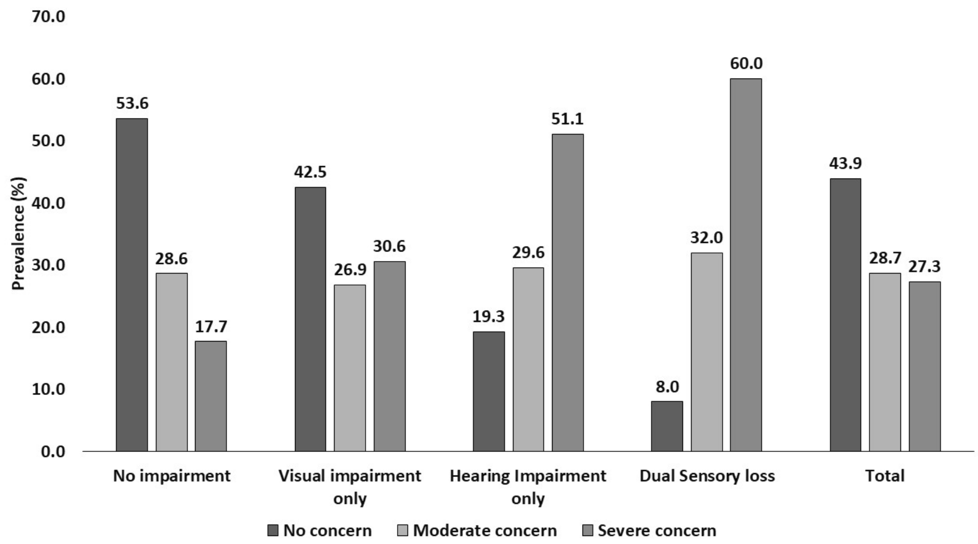

Sensory loss and fear of falling among elderly in residential care.

338x190mm (96 x 96 DPI)

STROBE 2007 (v4) Statement—Checklist of items that should be included in reports of *cross-sectional studies*

Section/Topic	Item #	Recommendation	Reported on page #
**Title and abstract**	1	(*a*) Indicate the study's design with a commonly used term in the title or the abstract	1
		(*b*) Provide in the abstract an informative and balanced summary of what was done and what was found	1
**Introduction**			
Background/rationale	2	Explain the scientific background and rationale for the investigation being reported	3
Objectives	3	State specific objectives, including any pre-specified hypotheses	3
**Methods**			
Study design	4	Present key elements of study design early in the paper	3
Setting	5	Describe the setting, locations, and relevant dates, including periods of recruitment, exposure, follow-up, and data collection	4,5
Participants	6	(*a*) Give the eligibility criteria, and the sources and methods of selection of participants	5,
Variables	7	Clearly define all outcomes, exposures, predictors, potential confounders, and effect modifiers. Give diagnostic criteria, if applicable	6,7
Data sources/ measurement	8*	For each variable of interest, give sources of data and details of methods of assessment (measurement). Describe comparability of assessment methods if there is more than one group	6,7
Bias	9	Describe any efforts to address potential sources of bias	NA
Study size	10	Explain how the study size was arrived at	5
Quantitative variables	11	Explain how quantitative variables were handled in the analyses. If applicable, describe which groupings were chosen and why	7
Statistical methods	12	(*a*) Describe all statistical methods, including those used to control for confounding	7
		(*b*) Describe any methods used to examine subgroups and interactions	7
		(*c*) Explain how missing data were addressed	NA
		(*d*) If applicable, describe analytical methods taking account of sampling strategy	NA
		(*e*) Describe any sensitivity analyses	NA
**Results**			

For peer review only

Participants	13*	(a) Report numbers of individuals at each stage of study—eg numbers potentially eligible, examined for eligibility, confirmed eligible, included in the study, completing follow-up, and analysed	NA
		(b) Give reasons for non-participation at each stage	NA
		(c) Consider use of a flow diagram	NA
Descriptive data	14*	(a) Give characteristics of study participants (eg demographic, clinical, social) and information on exposures and potential confounders	7
		(b) Indicate number of participants with missing data for each variable of interest	NA
Outcome data	15*	Report numbers of outcome events or summary measures	7
Main results	16	(*a*) Give unadjusted estimates and, if applicable, confounder-adjusted estimates and their precision (eg, 95% confidence interval). Make clear which confounders were adjusted for and why they were included	7
		(*b*) Report category boundaries when continuous variables were categorized	7
		(*c*) If relevant, consider translating estimates of relative risk into absolute risk for a meaningful time period	7
Other analyses	17	Report other analyses done—eg analyses of subgroups and interactions, and sensitivity analyses	7,8
**Discussion**			
Key results	18	Summarise key results with reference to study objectives	11,12
Limitations	19	Discuss limitations of the study, taking into account sources of potential bias or imprecision. Discuss both direction and magnitude of any potential bias	12,13
Interpretation	20	Give a cautious overall interpretation of results considering objectives, limitations, multiplicity of analyses, results from similar studies, and other relevant evidence	13
Generalisability	21	Discuss the generalisability (external validity) of the study results	13
**Other information**			
Funding	22	Give the source of funding and the role of the funders for the present study and, if applicable, for the original study on which the present article is based	14

*Give information separately for cases and controls in case-control studies and, if applicable, for exposed and unexposed groups in cohort and cross-sectional studies.

Note: An Explanation and Elaboration article discusses each checklist item and gives methodological background and published examples of transparent reporting. The STROBE checklist is best used in conjunction with this article (freely available on the Web sites of PLoS Medicine at http://www.plosmedicine.org/, Annals of Internal Medicine at http://www.annals.org/, and Epidemiology at http://www.epidem.com/). Information on the STROBE Initiative is available at www.strobe-statement.org.

For peer review only
